# Modeling within-level latent interaction effects in multilevel vector-autoregressive models

**DOI:** 10.3758/s13428-025-02694-3

**Published:** 2025-09-05

**Authors:** Jana Holtmann, Kenneth Koslowski

**Affiliations:** Wilhelm-Wundt Institute for Psychology, Leipzig University, Neumarkt 9-19, 04109 Leipzig, Germany

**Keywords:** Moderation, Latent interaction, Time series analysis, Dynamic structural equation modeling, Intensive longitudinal data

## Abstract

The study of time-dependent within-person dynamics has gained popularity in recent years through the use of multilevel (latent) time-series models. However, due to the complexity of the models, model applications are usually limited with respect to the inclusion of time-varying moderating factors on the longitudinal within-person relations between variables. That is, in common applications of multilevel time-series models, the within-person dynamics of constructs over time are regarded as being insensitive to changes in other time-varying factors or changes in contexts. We illustrate an extension of multilevel latent time-series models that incorporate latent interaction effects at the dynamic within-person level. We build on previous work that incorporated time-varying observed or latent moderator variables for the dynamic parameters in vector autoregressive models and provide a tutorial for the application of the models, implemented and estimated using Bayesian estimation via Markov chain Monte Carlo techniques. The models are illustrated by two empirical applications that investigate the temporal dynamics of negative affect, rumination, and mindful attention. The performance of different models with varying complexity is investigated via several simulation studies to provide recommendations regarding the models’ applicability for applied researchers. Required sample sizes range between at least 25 time points for around 50 persons in the simplest fixed-effects models and at least 100 time points for at least 100 persons in random-effects factor models, depending on the expected effect sizes of the dynamic parameters.

**Supplementary Information:**

The online version contains supplementary material available at 10.3758/s13428-025-02694-3.

In recent years, technological and methodological advances have facilitated the collection and analysis of intensive longitudinal data (ILD). The increased availability of ILD has fostered the investigation of time-dependent within-person dynamics, giving credit to the observation that psychological processes happen within the individual (Hamaker, 2012; Molenaar, 2004). For instance, the degree of an individual’s experienced positive and negative emotions may vary across time with the occurrence of emotion-triggering events, social contexts, or the individual’s use of emotion-regulation strategies (e.g., Brans et al., 2013; Koval & Kuppens, 2011). That is, many questions in behavioral research concern the within-person relationship between different variables over time, e.g., the question whether the use of emotion-regulation strategies influences subsequent affective experiences, and the degree to which these relationships differ between persons. To model the temporal dynamics in one or several variables over time, researchers oftentimes make use of time-series models, such as autoregressive (AR) or vector-autoregressive (VAR) models (Hamilton, 1994; Lütkepohl, 2005), and their multilevel extensions (e.g., Asparouhov et al., 2018; Jongerling et al., 2015). The latter allow for person-specific dynamic parameters with between-person heterogeneity modeled at the cluster level, making it possible to estimate population distributions for the parameters.

In this paper, we illustrate and provide guidance on the application of multilevel latent VAR models with latent interaction effects at the dynamic within-person level. The models allow researchers to model complex relationships between variables as they unfold across time within the individual, while using latent person-mean centering and controlling for measurement error in the observations. The models can for instance be applied to capture context-related change in the joint dynamics of latent constructs across time. These changes in the (joint) dynamics of latent constructs are induced by time-varying latent moderator variables at the within-person level.

In AR models, serial dependence in ILD is modeled by regressing a variable’s present value on temporally preceding values of the same variable. Thereby, the temporal dynamics are described by two parameters: the AR effect of the variable, also referred to as carryover effect or inertia (Kuppens et al., 2010; Suls et al., 1998), which quantifies the speed at which a process returns to its equilibrium after a deviation due to an external force on the system; and the dynamic residual, also referred to as innovation, with the variance of these innovations quantifying the degree of variability in the process that remains unexplained by preceding observations. In the multivariate VAR model, the joint dynamics of several variables are modeled, with the possibility to investigate potential reciprocal relationships across time. In VAR models, cross-regression (CR) effects between the variables capture the degree to which past values of a variable can predict future values of the other variable. The respective other variables thus serve as time-varying covariates that predict the subsequent level of a variable and may thereby change the mean of the process over time. In many cases, these covariates may capture contextual information in the form of a categorical covariate (e.g., the occurrence of certain external events/experienced situations) but also in the form of continuous covariates (e.g., the degree of experienced stress).

Besides changing the level of the dependent variable, covariates might also affect the dynamics of the dependent variable(s) over time, changing inertia, residual variability, or reciprocal effects between variables. For instance, stress was found to moderate emotional inertia (Koval & Kuppens, 2012) as well as the association between positive and negative affect over time (e.g., Zautra et al., 2002). To identify time-varying moderators of temporal dynamics as captured by AR and CR effects, researchers may include an interaction effect between a time-varying predictor and a covariate. In *N* = 1 (V)AR models or univariate multilevel models, this can be achieved by including lagged predictor–covariate product terms. However, specifying this kind of interaction effect is more intricate in multivariate multilevel VAR models. This is because these modeling approaches perform latent person-mean centering (see Asparouhov et al., 2018; Asparouhov & Muthén, 2019), resulting in the dynamics taking place at the level of within-person latent variables, with the respective need to calculate latent interaction effects. Currently, multivariate multilevel VAR models with latent within-level interaction effects are not integrated in ready-to-use software implementations (e.g., in Mplus), thereby limiting the models’ applicability for applied researchers, unless these are apt to dive into and adapt complex model codes to their needs. Therefore, researchers wishing to investigate interaction effects in multilevel time-series models have relied on observed mean-centered product terms (see, e.g., Bolzenkötter et al., 2024) or proposed two-step workarounds which rely on the extraction of factor scores (Speyer et al., 2024). Using observed person-mean centering does, however, come with the risk of biased estimates of autoregressive effects, known as Nickell's bias (Nickell, 1981).

Apart from incorporating the advantages of latent person-mean centering (e.g., resolving Nickell's bias; Asparouhov et al., 2018), modeling temporal dynamics based on latent variables is also advantageous in terms of controlling for measurement error in the observations, which is ubiquitous in the behavioral sciences. Critically, measurement error in observed time series is known to have detrimental effects on the estimation of individual AR parameters (Du & Wang, 2018; Koslowski & Holtmann, in press; Schuurman & Hamaker, 2019; Schuurman et al., 2015; Staudenmayer & Buonaccorsi, 2005). (Multilevel) VAR models can be extended to account for measurement error in the observed variables in single-indicator models (Schuurman & Hamaker, 2019; Schuurman et al., 2015), as well as by including a measurement model in the case of multiple available indicators per construct (Asparouhov et al., 2018). The latter models were termed dynamic structural equation models (DSEM) in Asparouhov et al. (2018) and are an extension of dynamic factor models (Molenaar, 1985; Zhang et al., 2008) to multilevel models for time series of several individuals. Here, we will illustrate the extension of these multilevel latent VAR models to include latent interaction effects at the dynamic within-person level.

Interaction effects in longitudinal models and the moderation of dynamic processes have received some attention in recent years. For instance, Ozkok et al. (2022) discuss both within-within-level and between-within-level interactions in (single-level) random-intercept cross-lagged panel models, where within and between refer to the time-specific and the stable level of observation, respectively. However, the models discussed by Ozkok et al. (2022) do not include a measurement model for the outcome or moderator variables, nor do they extend to multilevel models that are suitable for the analysis of ILD. Adolf et al. (2017) introduced fixed moderated time-series analysis, an approach that allows one to incorporate time-varying observed moderator variables for the dynamic parameters in *N* = 1 VAR models. Fixed moderated time-series analysis is a flexible approach that allows researchers to moderate not only AR and CR effects but also innovation variances, by an observed categorical or continuous moderator variable. However, Adolf et al. (2017) did not consider latent moderator or latent dependent variables and only considered the *N* = 1 case. Kelava and Brandt (2019) introduced nonlinear dynamic latent class structural equation modeling (NDLC-SEM). The NDLC-SEM framework incorporates a large class of models by extending dynamic latent class analysis (DLCA; Asparouhov et al., 2017), which combines latent Markov and dynamic SEM models (Asparouhov et al., 2018). NDLC-SEM extends DLCA to incorporate both inter-individual and time-specific random effects as well as nonlinear effects. Thereby, NDLC-SEM does, for instance, allow for heterogeneous trajectories as well as changes in the dynamic process over time as a result of unobserved entities modeled via latent states in a Markov switching process. The framework in its theoretical definition is very general and does cover latent interaction effects at both levels of observation.

We build on this previous work (Adolf et al., 2017; Kelava & Brandt, 2019) and provide a tutorial, a practical illustration, and a simulation study for multilevel latent VAR models with latent moderator and outcome variables. To date, the performance of multilevel VAR models with latent interaction effects at the dynamic within-person level has not been investigated in detail (e.g., in existing simulation studies on the NDLC-SEM framework; Andriamiarana et al., 2023), and recommendations on the required sample sizes and time-series lengths are still missing. In a comprehensive simulation study, we investigate the performance of the different model variants under different lengths of the time series and sample sizes, and derive recommendations for applications of the models. The approach is illustrated with two empirical applications to model the dynamic interplay of negative affect and rumination and its moderation by mindful attention.

The models are implemented using a Bayesian Markov chain Monte Carlo (MCMC) approach in the Stan software program (Carpenter et al., 2017). Fitting complex multilevel VAR models may be a challenge for researchers unfamiliar with Bayesian estimation or the specifics of MCMC software modeling languages. To make the models accessible to applied researchers, we provide model codes for all model variants along with accompanying R code for data preparation. The manuscript is accompanied by a tutorial that guides users through the model code and illustrates and explains the building blocks of both a fixed-effects and random-effects multilevel latent moderated VAR model; see Appendix D and the online supplemental material (OSM) on the project’s OSF page (https://osf.io/bvsqy).

## Multilevel time-series models with within-level latent interactions

We illustrate three different within-person dynamic interaction models as possible scenarios of models that might be interesting to answer substantive research questions. The models are sub-models of the NDLC-SEM framework (Kelava & Brandt, 2019), focusing on within-level latent interaction effects in VAR models. Note that (a) between-within-level interaction effects are easily accommodated in this kind of model (see, e.g., Asparouhov et al., 2018) by relating individual AR and CR parameters to external covariates at the between-person level, and (b) latent between-between-level interaction effects are covered and investigated elsewhere (see, e.g., Asparouhov & Muthén, 2021; Andriamiarana et al., 2023). The models accommodate up to three latent variables at both the within- and between-person levels. Note that these models are examples of possible time-series interaction models, which can be easily adapted to different dynamics and interaction effects between the variables to match a respective substantive research question. Furthermore, the within-person time-series models can be flexibly combined with different measurement models and fixed- and random-effects components of the multilevel structure, resulting in models of varying complexity. The simplest of these models is a fixed-effects multilevel VAR model that includes interaction effects at the within-person level of two latent person-mean-centered variables across time. This model can be extended to include several indicators per construct, with measurement models for all variables at both the within- and between-person levels, resulting in the within-person dynamic process and interaction effects unfolding at the level of latent factors. The models can be further extended to accommodate person-specific dynamics by modeling AR, CR, and interaction effects, as well as innovation (co-)variances as random effects. Note that all of the presented models assume random-mean variables—that is, the average (latent) levels of the variables, conceptualized as stable latent trait variables, are always considered to be person-specific and normally distributed across persons. The presentation of the models is structured into four subsections: (a) decomposition of the observed variables and measurement models, (b) within-level dynamic interaction models, (c) the special case of random innovation covariances, and (d) the between-level structural model.

### Decomposition and measurement models

As a working example, consider the joint dynamics of momentary mindful attention (MF; ${\eta }_{1}$), negative affect (NA; ${\eta }_{2}$), and rumination (RU; ${\eta }_{3}$) across time. We assume that $Q$ (here: $Q\le 3)$ latent variables ${\eta }_{qit}$ ($q=1,\dots ,Q$) are measured by *P* observed indicators ${Y}_{pit}$ ($p=1,\dots ,P$) for $i=1,\dots ,N$ individuals at $t=1,\dots ,T$ time points. That is, several constructs are measured across time, with time points (time-specific within-person level) clustered within persons (between-person level). Observed indicator variables are decomposed into their respective between- and within-level components by latent person-mean centering,

1 $${{\varvec{Y}}}_{it}= {{\varvec{\mu}}}_{i}+{{\varvec{Y}}}_{it}^{w}$$

where ${{\varvec{Y}}}_{it}$ is a $(P\times 1)$-dimensional vector of observed indicator variables for person *i* at time *t*, ${{\varvec{\mu}}}_{i}$ is a ($P\times 1)$ vector of latent person means (random intercepts), and ${{\varvec{Y}}}_{it}^{w}$ is a ($P\times 1)$ vector of within-level latent person-mean-centered, time-specific deviations. At both levels of observation, that is, for the latent trait variables ${{\varvec{\mu}}}_{i}$ and the time-specific state variables ${{\varvec{Y}}}_{it}^{w}$, we can include a measurement model for the definition of a common latent stable trait factor and a common time-specific latent state factor, respectively. The respective measurement models are given by

2 $${{\varvec{Y}}}_{it}^{w}={{\varvec{\lambda}}}_{W} {{\varvec{\eta}}}_{it}^{w}+{{\varvec{\varepsilon}}}_{it}^{w}$$

3 $${{\varvec{\mu}}}_{i}=\boldsymbol{\alpha }+ {{\varvec{\lambda}}}_{B} {{\varvec{\eta}}}_{i}^{\mu }+{{\varvec{\varepsilon}}}_{i}^{B}$$

where ${{\varvec{\lambda}}}_{W}$ and ${{\varvec{\lambda}}}_{B}$ are ($P\times Q)$-dimensional matrices of factor loadings, $\boldsymbol{\alpha }$ is a $(P\times 1)$-dimensional vector of item intercept parameters, and ${{\varvec{\varepsilon}}}_{it}^{w}$ and ${{\varvec{\varepsilon}}}_{i}^{B}$ are ($P\times 1)$ vectors of serially uncorrelated measurement residuals, which are assumed to be independently and identically distributed (*iid*) as ${{\varvec{\varepsilon}}}_{it}^{w}\sim N(0,{{\varvec{\Theta}}}_{w})$ and ${{\varvec{\varepsilon}}}_{i}^{B}\sim N\left(0,{{\varvec{\Theta}}}_{B}\right)$, with ${{\varvec{\Theta}}}_{w}$ and ${{\varvec{\Theta}}}_{B}$ being diagonal matrices of dimension $(P\times P)$. The vectors ${{\varvec{\eta}}}_{it}^{w}$ and ${{\varvec{\eta}}}_{i}^{\mu }$ contain the *Q* latent time-specific state factors and stable latent trait factors, respectively. For ease of model presentation, we assume that the number of latent factors in Eqs. 2 and 3 is constant across levels. However, this is not necessary in practice, and the between-level measurement model could, for instance, be modified to model item-specific stable trait effects instead of a common factor (see, e.g., Geiser et al., 2013).

**Fig. 1:**
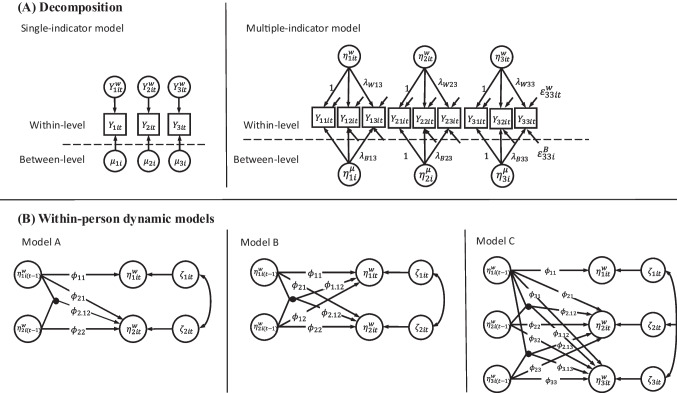
Graphical representation of different multilevel latent within-level interaction time-series models. **A** Decomposition of observed variables into between- and within-level components, in the single-indicator case (left-hand side) and multiple-indicator case (latent factor model; right-hand side). **B** Within-level latent interaction models; note that in the single-indicator case (see panel **A** left-hand side), the latent variable ${\eta }_{it}^{w}$ is identical to the respective ${Y}_{it}^{w}$

In the case of a single-indicator model, which does not include measurement models for latent factors at the within- and between-person levels, the respective factor loadings in Eqs. 2 and 3 are set to ${{\varvec{\lambda}}}_{W}=1$ and ${{\varvec{\lambda}}}_{B}=1$ and the measurement error terms ${{\varvec{\varepsilon}}}_{it}^{w}$ and ${{\varvec{\varepsilon}}}_{i}^{B}$ are not modeled, resulting in ${{\varvec{Y}}}_{it}^{w}={{\varvec{\eta}}}_{it}^{w}$ and ${{\varvec{\mu}}}_{i}= {{\varvec{\eta}}}_{i}^{\mu }$. See Fig. 1A for a graphical depiction of the single- and multiple-indicator model variants. Note that it is also theoretically possible to model the within-level error terms ${{\varvec{\varepsilon}}}_{it}^{w}$ in single-indicator time-series models (see, e.g., Schuurman et al., 2015; Schuurman & Hamaker, 2019). The need to separate measurement error and dynamic error in these models based solely on the time-series dynamics, does, however, increase model complexity and estimation demands. Therefore, we will not further consider this specific kind of model in the following.

### Within-level VAR models with latent interactions

We exemplify three different VAR(1) models with latent interaction effects at the dynamic within-person level. In all of the following models, the serial dependency in the covariate/moderator variable across time is considered by the inclusion of an AR(1) process for the respective variable. Consider, for example, the previous level of MF $\left({\eta }_{1}\right)$ predicting the subsequent level of NA (${\eta }_{2}$) as well as the autoregression in NA. In this model, MF can be considered as moderating the degree to which NA persists over time. Alternatively, the interaction could be conceptualized as a moderation of the degree to which MF can improve NA by the previous level of NA itself. This model is graphically depicted as Model A in Fig. 1B and is a special case of the two-level nonlinear dynamic latent class model presented in Kelava and Brandt (2019). The model is defined by

4 $${\eta }_{1it}^{w}= {\phi }_{11i}{\eta }_{1i\left(t-1\right)}^{w}+{\zeta }_{1it}$$

5 $${\eta }_{2it}^{w}= {\phi }_{21i}{\eta }_{1i\left(t-1\right)}^{w}+{\phi }_{22i}{\eta }_{2i\left(t-1\right)}^{w}+ {\phi }_{2.12i}({\eta }_{1i\left(t-1\right)}^{w}{*{\eta }_{2i\left(t-1\right)}^{w})+ \zeta }_{2it}$$

where the parameters ${\phi }_{qq{\prime}i}$ denote the AR and CR effects of construct $q{\prime}$ at time $(t-1)$ on construct $q$ at time $t$, and ${\phi }_{k.qq{\prime}i}$ denotes the interaction effect of constructs $q$ and $q{\prime}$ at time $\left(t-1\right)$ on construct $k$ at time $t$. AR parameters quantify the degree of a constructs’ persistence over subsequent time points (carryover effect or inertia). The closer the AR effect is to zero, the faster the constructs’ return to baseline levels once deviated to higher or lower levels. CR effects capture predictive relationships, that is, reciprocal effects between the different constructs over time (controlling for the autoregression). Because of the multilevel data structure, all effects refer to levels relative to the respective persons’ average trait levels. The variables ${\zeta }_{qit}$ denote latent dynamic regression residuals, also termed innovations, which are assumed to be serially uncorrelated across time (iid across time points), with a multivariate normal distribution with mean zero, variances ${\sigma }_{{\zeta }_{1i}}^{2}$ and ${\sigma }_{{\zeta }_{2i}}^{2}$, and covariance ${\sigma }_{{\zeta }_{12i}}$ within time points

6 $$\left[\begin{array}{c}{\zeta }_{1it}\\ {\zeta }_{2it}\end{array}\right] \sim MVN\left(\begin{array}{cc}\left[\begin{array}{c}0\\ 0\end{array}\right],& \left[\begin{array}{cc}{\sigma }_{{\zeta }_{1i}}^{2}& {\sigma }_{{\zeta }_{12i}}\\ {\sigma }_{{\zeta }_{12i}}& {\sigma }_{{\zeta }_{2i}}^{2}\end{array}\right]\end{array}\right)$$

Innovations capture unexplained fluctuations across time, that is, time-specific deviations in the constructs that remain unexplained by previous within-level states included in the model. They are dynamic in the sense that they are carried forward in time, impacting subsequent observations. The innovations’ variances capture the instability of a process, while the within-time-point correlation of two constructs’ innovations quantifies how far unobserved external factors occurring at time $t$ exert an impact on the systems of both constructs to the same degree, in the same or opposite directions.

The model can be extended by considering a fully crossed predictive structure across time between the two variables. That is, we assume that previous levels of NA also have an impact on the degree to which MF is practiced in the following time period. In addition, MF and NA might both depend on the combination of previous levels of MF and NA, that is, their interaction effect. The respective model is depicted as Model B in Fig. 1B and defined by

7 $${\eta }_{1it}^{w}= {\phi }_{11i}{\eta }_{1i\left(t-1\right)}^{w}+{\phi }_{12i}{\eta }_{2i\left(t-1\right)}^{w}+ {\phi }_{1.12i}({\eta }_{1i\left(t-1\right)}^{w}*{\eta }_{2i\left(t-1\right)}^{w}) +{\zeta }_{1it}$$

8 $${\eta }_{2it}^{w}= {\phi }_{21i}{\eta }_{1i\left(t-1\right)}^{w}+{\phi }_{22i}{\eta }_{2i\left(t-1\right)}^{w}+ {\phi }_{2.12i}({\eta }_{1i\left(t-1\right)}^{w}{*{\eta }_{2i\left(t-1\right)}^{w})+ \zeta }_{2it}$$

Innovation variances are assumed to follow the same distribution as given in Eq. 6.

The third model that we consider is a within-level VAR model including three latent variables, depicted as Model C in Fig. 1B. Consider a fully crossed VAR structure between NA (${\eta }_{2}$) and RU (${\eta }_{3}$) across time. That is, we assume that NA and RU both show some carryover effects across time and both depend on the respective other constructs’ previous levels, with a potentially positive correlation of the constructs’ innovations within time points. These temporal relationships between NA and RU are modeled as a function of MF (${\eta }_{1}$). That is, the degree to which an individual employs MF is assumed to predict subsequent levels of both RU and NA as well as their temporal relationship over time. The serial dependency in MF is modeled via an AR(1) structure. The respective model is given by

9 $${\eta }_{1it}^{w}={\phi }_{11i} {\eta }_{1i\left(t-1\right)}^{w}+{\zeta }_{1it}$$

10 $$\begin{array}{l}{\eta }_{2it}^{w}= {\phi }_{21i}{\eta }_{1i\left(t-1\right)}^{w}+{\phi }_{22i}{\eta }_{2i\left(t-1\right)}^{w}+ {\phi }_{23i}{\eta }_{3i\left(t-1\right)}^{w}\\ + {\phi }_{2.13i}\left({\eta }_{1i\left(t-1\right)}^{w}*{\eta }_{3i\left(t-1\right)}^{w}\right)+ {\phi }_{2.12i}\left({\eta }_{1i\left(t-1\right)}^{w}*{\eta }_{2i\left(t-1\right)}^{w}\right)+{\zeta }_{2it}\end{array}$$

11 $$\begin{array}{l}{\eta }_{3it}^{w}= {\phi }_{31i}{\eta }_{1i\left(t-1\right)}^{w}+{\phi }_{32i}{\eta }_{2i\left(t-1\right)}^{w}+ {\phi }_{33i}{\eta }_{3i\left(t-1\right)}^{w}\\ + {\phi }_{3.13i}\left({\eta }_{1i\left(t-1\right)}^{w}*{\eta }_{3i\left(t-1\right)}^{w}\right)+{\phi }_{3.12i}\left({\eta }_{1i\left(t-1\right)}^{w}*{\eta }_{2i\left(t-1\right)}^{w}\right)+{\zeta }_{3it}\end{array}$$

In this example, ${\eta }_{1}$ at time $t-1$ moderates the AR and CR effects between ${\eta }_{2}$ and ${\eta }_{3}$ across time. The model could be extended by a moderating effect of MF on the innovation variances and covariances of NA and RU.

### Innovation covariances in random-effects models

Equation 6 defines the joint distribution of the innovations within time points. In the case of fixed-effects models, the respective innovation variances and covariance are assumed to be constant across individuals and the index $i$ is dropped, resulting in the (co)variance terms ${\sigma }_{{\zeta }_{1}}^{2}$, ${\sigma }_{{\zeta }_{2}}^{2}$, and ${\sigma }_{{\zeta }_{12}}$. In this case, the innovations can be easily modeled via a multivariate normal distribution. In the case of random (co)variances, which receive a distribution across persons with a respective average level (fixed-effect) and (random-effect) variance, implementation via a multivariate normal distribution at the within-person level is possible (see, e.g., Li et al., 2022), although difficult to implement considering sampling efficiency (i.e., using vectorization and Cholesky factors of correlation matrices). In complex models, such as the latent moderated VAR models presented herein, it is essential to consider sampling efficiency, as a lack of it can render the sampling tediously slow. We therefore follow an approach suggested by Hamaker et al. (2018), modeling the person-specific innovation covariances by introducing a new, common factor ${\eta }_{\zeta it}$, with residuals ${\delta }_{1it}$ and ${\delta }_{2it}$ (see Appendix A for details). The variances of the respective variables are assumed to be log-normally distributed at the between-person level (i.e., modeling $\text{ln(}\sigma_{\eta\zeta i}^2),\text{ln(}\sigma_{\delta1i}^2)$, and ${\text{ln(}\sigma}_{\delta2i}^2)$ as (multivariate) normally distributed). The reparameterization is primarily relevant for setting up the model code (see tutorial in Appendix D and the Stan codes in the OSM) and is, for this purpose, explained in detail in Appendix A. Note, however, that this parameterization assumes that the innovation covariances are of the same sign for all individuals.

### Between-level model (random-effect covariances)

In the within-level VAR models defined in Eqs. 4–11, AR, CR, and interaction effects, as well as innovation (co)variances, carry an index $i$, denoting that these parameters can be modeled as person-specific. Thereby, the model can account for inter-individual differences in the latent within-person dynamics over time. However, modeling all parameters as random effects increases model complexity substantially and thus also estimation demands and required sample sizes (see, e.g., Asparouhov et al., 2018; Schultzberg & Muthén, 2018). Therefore, in small samples or in the absence of substantial between-person heterogeneity in the respective effects, parameters can be fixed to be equal across persons (see, e.g., Asparouhov & Muthén, 2022, for recommendations).

In the case of assuming fixed parameters of the within-person VAR model, the between-level covariance matrix reduces to the (co)variances of the latent trait variables (latent person averages), with

12 $$\left[\begin{array}{c}{\eta }_{1i}^{\mu }\\ {\eta }_{2i}^{\mu }\end{array}\right] \sim MVN\left(\left[\begin{array}{c}{\gamma }_{\mu 1}\\ {\gamma }_{\mu 2}\end{array}\right]\begin{array}{cc},& \left[\begin{array}{cc}{\tau }_{{\mu }_{1}}^{2}& \\ {\tau }_{{\mu }_{1},{\mu }_{2}}& {\tau }_{{\mu }_{2}}^{2}\end{array}\right]\end{array}\right)$$

for the case of $Q=2$ latent trait factors.

In the random-effects model, the within-level VAR parameters ${\phi }_{qqi}$, ${\phi }_{qq{\prime}i}$, ${\phi }_{k.qq{\prime}i}$, $\text{ln}({\sigma }_{\eta \zeta i}^{2})$, and ${\text{ln}(\sigma }_{\delta qi}^{2})$ are also assumed to follow a multivariate normal distribution, with means ${\gamma }_{{\phi }_{qq}}$, ${\gamma }_{{\phi }_{qq{\prime}}}$, ${\gamma }_{{\phi }_{k.qq{\prime}}}$, ${\gamma }_{\sigma \eta \zeta }$, and ${\gamma }_{\sigma \delta q}$ (average effects across persons/fixed effects) and respective variances of the person-specific deviations from these average effects, ${\tau }_{{\phi }_{qq}}^{2}$, ${\tau }_{{\phi }_{qq{\prime}} }^{2}$, ${\tau }_{{\phi }_{k.qq{\prime}}}^{2}$, ${\tau }_{\sigma \eta \zeta }^{2}$, ${\tau }_{\sigma \delta q}^{2}$ (variation across individuals; random effects). In addition, covariances between the person-specific parameters can be modeled, for instance, to investigate whether higher trait levels of RU are associated with higher trait NA or with higher predictive effects of RU on NA.

## Model implementations

The models described above were implemented using a Bayesian MCMC approach in the free software Stan (Carpenter et al., 2017). To facilitate the adaptation of the provided Stan model codes to within-level VAR and interaction models not directly covered by these three exemplary models, different sections of the Stan model codes are described and discussed in detail in the accompanying tutorial provided in Appendix D. In the code snippets described in Appendix D, model code is held as general as possible (with respect to the number of indicator variables per factor, the number of within-level regression parameters, etc.). In addition, the model codes include the computation of standardized within-level regression parameters. In the case of multilevel VAR models, within-person standardization is recommended and implemented for CR effects (Schuurman et al., 2016a). The computation of these standardized effects is described in detail in Appendix A. Accompanying R code, provided on OSF, illustrates the data preparation steps as well as the generation of interaction plots based on the parameters’ posterior samples (see empirical examples).

## Empirical examples: Moderated affect dynamics

We illustrate the approach to multilevel latent moderated VAR modeling with two applications to the dynamic interplay of NA, RU, and MF. Mindfulness as an emotion regulation strategy, defined by paying attention to and appreciating one’s thoughts and emotions without evaluating or changing them, has gained increased attention in recent years (e.g., Blanke et al., 2020; Bolzenkötter et al., 2024; van der Gucht et al., 2019; Wenzel et al., 2023). Applying mindfulness has been linked with greater emotion differentiation and with changes in negative affect, positive affect, and rumination (e.g., Blanke et al., 2020; Bolzenkötter et al., 2024; van der Gucht et al., 2019; Wenzel et al., 2023). The maladaptive emotion regulation strategy of rumination is characterized by uncontrollable and repetitive negative thinking (Smith & Alloy, 2009) and is assumed to be a risk factor for psychopathology (Nolen-Hoeksema et al., 2008). Rumination was found to show dynamical associations with NA which manifest as mutually reinforcing and self-reinforcing spirals (Blanke et al., 2022; Moberly & Watkins, 2008; Selby et al., 2016). Recent findings suggest that mindfulness may not only reduce both NA and RU (Blanke et al., 2020; Bolzenkötter et al., 2024) but also impact the temporal dynamics of NA (Rowland et al., 2020) as well as the relationship between NA and RU (Blanke et al., 2020). In the current empirical examples, we perform reanalysis for the aforementioned selected variables of (1) one of the datasets used in Blanke et al. (2020) and (2) a dataset by Houben et al. (2023). In the first empirical example, we replicate the findings reported by Blanke et al. (2020) regarding the interaction effect between MF and RU on NA and extend the previous analyses by investigating the moderating effect of MF on the temporal link between RU and NA in a fully crossed VAR(1) model. In the second empirical example, we focus on the reciprocal relation between NA and RU and the question of whether the inertia of NA might be moderated by RU and whether this moderation effect varies across individuals.

### Samples, scales, and estimation

The first dataset stems from the study by Blanke et al. (2020) and is publicly available at OSF (https://osf.io/nvt6a/). The sample comprises 70 students who responded to ambulatory assessment (AA) items six times a day, within a time frame of 12 h, across a period of 9 days. On average, participants provided data on 54.4 time points (median = 54, *SD* = 3.25, min = 48, max = 65). The MF facet present-moment attention was measured using three items from the Multidimensional State Mindfulness Questionnaire (Blanke & Brose, 2017), RU was measured using the two items “I could not stop thinking about my feelings” and “I could not stop thinking about certain things,” and NA was assessed with three items (nervous, downhearted, distressed), each on a seven-point scale (see Blanke et al., 2020, for further details). All items were answered with a reference time frame of the time since the last beep (or since participants woke up in the morning).^1^

The second dataset stems from the study by Houben et al. (2023) and was made publicly available at https://osf.io/vt2q5/. The data comprise observations from 202 individuals collected in ambulatory assessments in three waves, with each wave comprising 10 beeps per day (distributed across a day between 10 a.m. and 10 p.m.) across a period of 7 days. In the following analyses, we used the data from all three waves jointly, excluding AR effects between observations from different waves. On average, participants provided data on 199 time points (median = 209, *SD* = 35, min = 69, max = 234). Participants answered five NA items (sad, angry, anxious, depressed, stressed), referring to their current level of experienced NA, and two rumination items (“Since the last beep, have you ruminated about something in the past/future?”) on a scale ranging from 0 to 100.

All models were estimated using four MCMC chains, with a chain length that was based on visual inspection of trace plots and the convergence criteria of Rhat < 1.01 and bulk and tail effective sample sizes (ESS) of > 100 per chain. The resulting chain length was 5,000 iterations for the models in empirical example 1 and 8,000 iterations in empirical example 2. All Stan model codes along with accompanying R code for the data preparation, model fitting, and analyses of the results are provided on OSF. We accounted for missed beeps and nighttime interruptions by modeling the former as missing values within the MCMC algorithm and the latter by excluding AR and CR effects from the last observation at night to the next observation in the morning (see tutorial in Appendix D).^2^

### Empirical example 1: Data by Blanke et al. (2020)

In light of the comparatively low number of available time points per person, in the current illustrative example we assume fixed within-level VAR parameters across individuals. The models include a measurement model for each of the three included constructs, with item-specific stable residual trait variables at the between-person level. Loading parameters of RU (with only two indicator items) were fixed to 1. In a first step (Model 1), we use the within-person model as reported by Blanke et al. (2020), which is extended to include AR effects for all constructs. In this model, NA is regressed on RU, MF, and their interaction, as assessed at the same time point, that is, referring to the same time period (see Fig. A2 in Appendix B for an exact graphical representation). We then fit Model C as described above, in which the AR and CR effects between RU and NA are moderated by previous MF. In this model (Model 2), the moderated effects are lagged effects (see Fig. A3 in Appendix B). In this way, in contrast to the simultaneous effect used in Model 1 and in Blanke et al. (2020), CR and interaction effects are expected to be of smaller magnitude due to the comparatively larger time distance between the measurement and the reference time frames of the variables.

**Fig. 2:**
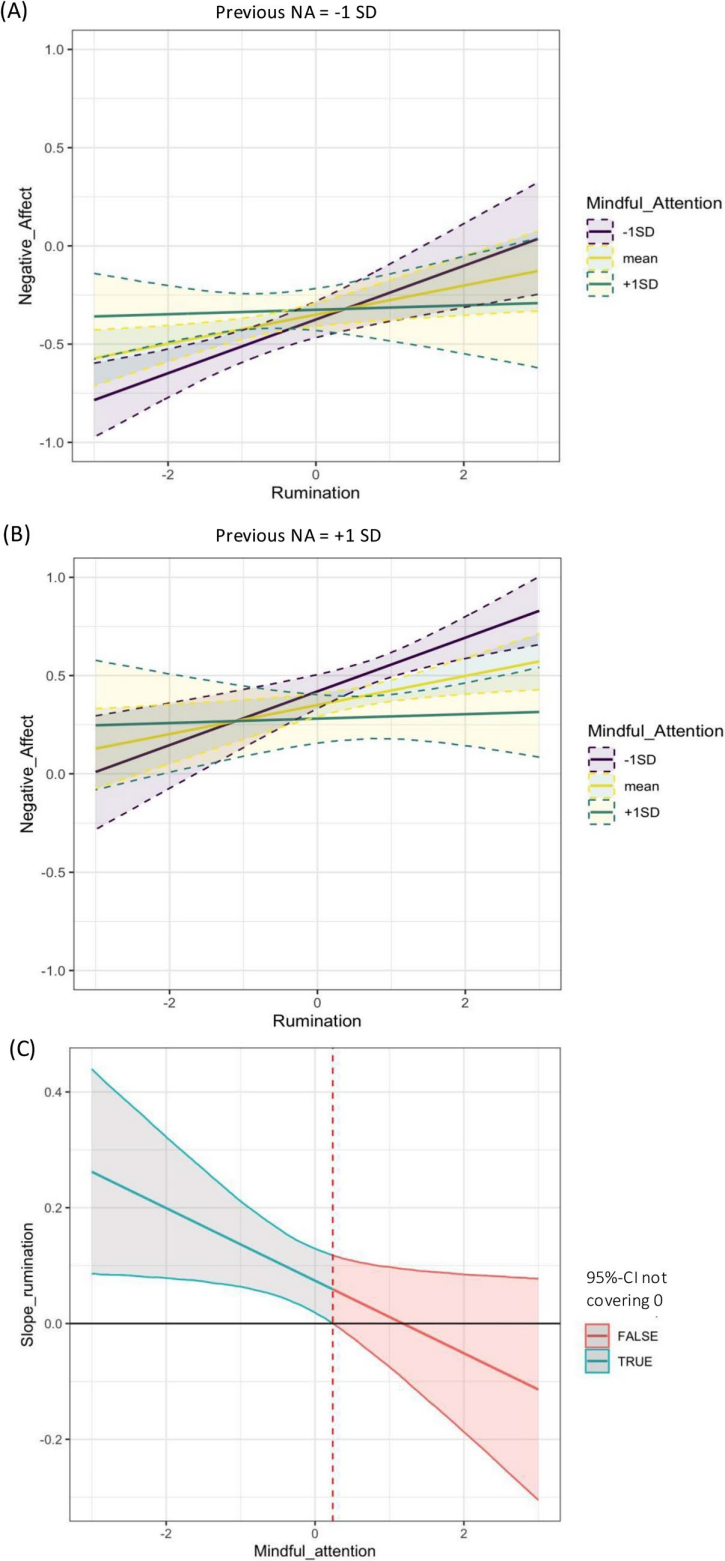
Interaction plots for empirical example 1, Model 2, using the data by Blanke et al. (2020). **A**, **B** Moderation of the effect of rumination (RU) at time $(t-1)$ on negative affect (NA) at time $t$ by previous mindful attention (MF) $(t-1)$, dependent on previous NA $(t-1)$. In panel **A**, previous NA is fixed at one standard deviation (*SD*) below the average, and in panel **B** at one *SD* above the average. The moderation is illustrated for three exemplary values of previous mindful attention, i.e., average previous MF (= 0), and one *SD* below and above the average. Solid lines represent the average predicted NA, and dashed lines depict the 95% credibility interval (CI) of the predicted NA values across posterior samples. **C** Model-implied slope of RU (as a predictor of NA) dependent on previous MF. The solid line represents the average predicted slope of RU given previous MF, and the surrounding shaded band depicts values that lie within the 95% CI of the predicted slope across posterior samples. Areas on the *x*-axis for which the shaded band is colored in red denote the values of previous MF for which the 95% CI of the slope covers zero (here for values of MF > 0.238)

**Fig. 3:**
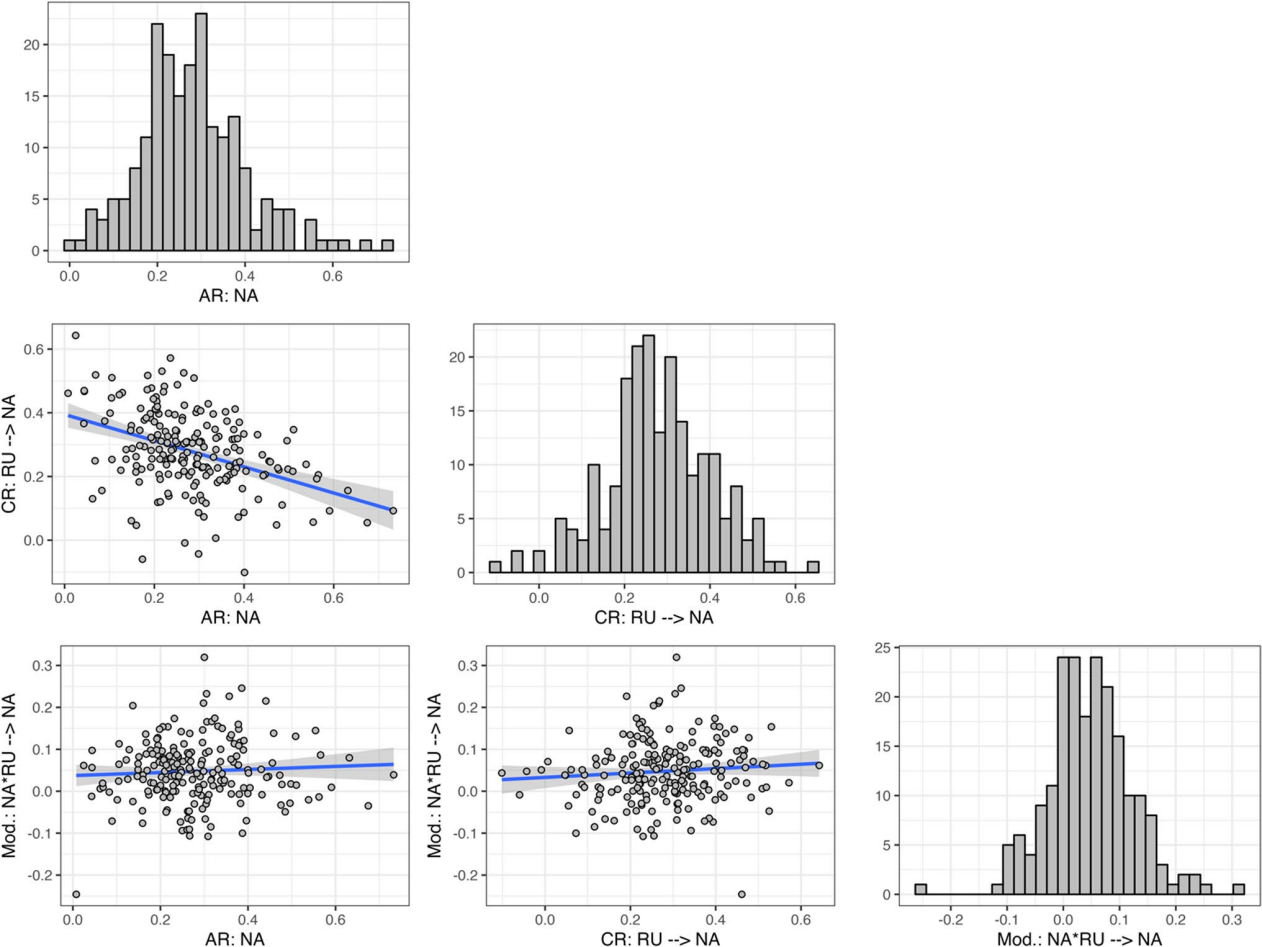
Histograms and scatterplots showing the distribution of individual-specific random-effect parameter estimates for the data application to the data by Houben et al. (2023). Displayed are within-person standardized parameter estimates for the autoregressive effect of negative affect, the cross-regression effect of rumination on subsequent negative affect, and the interaction/moderation effect of rumination and negative affect on subsequent negative affect. AR: autoregressive effect; CR: cross-regressive effect; Mod.: moderation effect; NA: negative affect; RU: rumination

Parameter estimates (posterior means) along with 95% credibility intervals (CI) for all model parameters of the models are provided in Tables A1–A4 in Appendix B. Estimates of the within-level moderated VAR model parameters are provided in Table 1. Results for Model 1 replicate the results reported in Blanke et al. (2020). We observe that MF is associated with a decrease in NA, while RU is associated with a comparatively greater increase in NA within the same time period. MF and RU show a negative interaction effect on NA, suggesting that the use of MF substantially decreases the negative effect of RU on NA. The interaction effect is larger than the one reported in Blanke et al. (2020).^3^ Together, RU and MF and the AR effect explain 55.7% (95% CI [50.27; 61.16]) of the within-level true-score variance in NA. Model-implied within-level reliabilities of the items range between 0.51 and 0.61 for RU, between 0.11 and 0.69 for NA, and between 0.32 and 0.66 for MF.

**Table 1:** Within-level parameter estimates of the multilevel latent moderated VAR model for mindfulness, rumination, and negative affect in the dataset by Blanke et al. (2020)

Parameter	Model 1 Blanke data				Model 2 Blanke data			
	Meaning	Estimate	95% CI	Std. est	Meaning	Estimate	95% CI	Std. Est
${\phi }_{11}$	${MF}_{(t-1)}$ → ${MF}_{t}$	**0.247**	[0.202; 0.293]	**0.247**	${MF}_{(t-1)} \to {MF}_{t}$	**0.238**	[0.192; 0.284]	**0.238**
${\phi }_{22}$	${NA}_{(t-1)} \to {NA}_{t}$	**0.230**	[0.187; 0.273]	**0.230**	${NA}_{(t-1)}$ → ${NA}_{t}$	**0.350**	[0.290; 0.409]	**0.350**
${\phi }_{33}$	${RU}_{(t-1)}$ → ${RU}_{t}$	**0.346**	[0.299; 0.393]	**0.346**	${RU}_{(t-1)} \to {RU}_{t}$	**0.231**	[0.170; 0.294]	**0.231**
${\phi }_{32}$	-	-	-	-	${NA}_{(t-1)} \to {RU}_{t}$	**0.133**	[0.071; 0.195]	**0.127**
${\phi }_{23}$	${RU}_{t}$ → ${NA}_{t}$	**0.474**	[0.423; 0.527]	**0.491**	${RU}_{(t-1)}$ → ${NA}_{t}$	**0.071**	[0.018; 0.124]	**0.074**
${\phi }_{21}$	${MF}_{t}$ → ${NA}_{t}$	** − 0.203**	[− 0.246; − 0.159]	** − 0.226**	${MF}_{(t-1)}$ → ${NA}_{t}$	− 0.020	[− 0.059; 0.020]	− 0.022
${\phi }_{31}$	-	-	-	-	${MF}_{(t-1)}$ → ${RU}_{t}$	** − 0.045**	[− 0.087; − 0.002]	** − 0.048**
${\phi }_{3.13}$	-	-	-	-	${MF}_{(t-1)}*{RU}_{(t-1)}$ → ${RU}_{t}$	− 0.018	[− 0.077; 0.041]	− 0.020
${\phi }_{3.12}$	-	-	-	-	${MF}_{(t-1)}*{NA}_{(t-1)}$ → ${RU}_{t}$	− 0.039	[− 0.100; 0.020]	− 0.041
${\phi }_{2.13}$	${MF}_{t}*{RU}_{t}$ → ${NA}_{t}$	** − 0.317**	[− 0.388; − 0.254]	** − 0.359**	${MF}_{(t-1)}*{RU}_{(t-1)}$ → ${NA}_{t}$	** − 0.055**	[− 0.106; − 0.004]	** − 0.063**
${\phi }_{2.12}$	-	-	-	-	${MF}_{(t-1)}*{NA}_{(t-1)}$ → ${NA}_{t}$	− 0.043	[− 0.110; 0.023]	− 0.047
${\sigma }_{{\zeta }_{1}}$	Innov. *SD* MF	0.949	[0.908; 0.991]	-	Innov. *SD* MF	0.962	[0.922; 1.004]	-
${\sigma }_{{\zeta }_{2}}$	Innov. *SD* NA	0.548	[0.503; 0.592]	-	Innov. *SD* NA	0.781	[0.747; 0.814]	-
${\sigma }_{{\zeta }_{3}}$	Innov. *SD* RU	0.821	[0.784; 0.858]	-	Innov. *SD* RU	0.815	[0.779; 0.851]	-
${r(\zeta }_{1},{\zeta }_{2})$	-	-	-	-	Innov. cor. MF-NA	** −.324**	[−.373; −.274]	-
${r(\zeta }_{1},{\zeta }_{3})$	-	-	-	-	Innov. cor. MF-RU	** −.294**	[−.345; −.241]	-
${r(\zeta }_{2},{\zeta }_{3})$	-	-	-	-	Innov. cor. RU-NA	**.575**	[.528;.622]	-

Estimates refer to the posterior mean values. 95% Credibility intervals (CI) are given by the 2.5% and 97.5% quantiles of the respective parameter’s posterior distribution. Regression coefficients with a 95% CI not covering zero are printed in boldface. Std. est.: standardized parameter estimates; MF: mindful attention; NA: negative affect; RU: rumination; *SD*: standard deviation; Innov.: Innovation; cor.: correlation

Results for Model 2 suggest that applying the emotion regulation strategy mindful attention is associated with lower levels of rumination in the following time period (${MF}_{(t-1)}$ → ${RU}_{t}$;

${\phi }_{31}= -0.045$), while high levels of NA predict subsequently higher levels of rumination (${NA}_{(t-1)}$ → ${RU}_{t}$; ${\phi }_{32}=0.133$). With respect to the prediction of NA in the fully lagged model, MF in the previous time period does not substantially predict NA in the following time period (${MF}_{(t-1)}$ → ${NA}_{t}$; n.s.), while rumination is associated with increased NA in the following time period (${RU}_{(t-1)}$ → ${NA}_{t}$;

${\phi }_{23}= 0.071$). This effect of previous RU on subsequent NA is moderated by the level of previous MF (${MF}_{(t-1)}*{RU}_{(t-1)}$ → ${NA}_{t}$; ${\phi }_{2.13}=-0.055$). That is, the use of MF is associated with a diminished effect of RU on subsequent NA. All remaining interaction effects were not substantially different from zero. Note that the effects are smaller than those in Model 1 due to the prolonged time interval between the measurements. The simultaneous effects of Model 1 are reflected in the innovation correlations of NA, RU, and MF in Model 2, which are in line with the results reported above.

The interaction effect of MF with previous RU on subsequent NA is illustrated via an interaction plot in Fig. 2. Parameter estimates for the simple slopes are generated using standardized parameter estimates of the AR, CR, and interaction effects. The predicted values of the dependent latent variable are calculated per MCMC iteration for selected values of the standardized moderator variable across a plausible range of the predictor variable. Interaction plots depict the average predicted value of NA for each (selected) moderator–predictor combination across post-burn-in iterations (solid line) and the 2.5% and 97.5% quantiles of the predicted values across MCMC iterations (shaded areas).

Figure 2C depicts the predicted standardized CR coefficient of RU on subsequent NA as a function of previous MF. The predicted coefficient is calculated as the average and the 95% CI as the 2.5% and 97.5% quantiles of the predicted slope across MCMC iterations. Areas of the 95% CI covering zero are depicted in red, and areas where the 95% CI does not cover zero are depicted in blue, with the latter showing the predictor range where the slope can be expected to be substantially different from zero. Exemplary R codes for generating the plots are provided on OSF.

### Empirical example 2: Data by Houben et al. (2023)

With an average of 199 time points per person, the dataset by Houben et al. (2023) is well suited for illustrating a random-effects model with person-specific interaction effects (see results of the following simulation studies for sample size recommendations). In this empirical example, we investigate the temporal dynamics of NA and RU and potential between-person differences in their reciprocal effects. For this purpose, we use a slightly modified variant of Model B as described above. As the items for RU were formulated to capture the degree to which participants practiced RU since the last AA signal, we used RU as reported at the same measurement time point (referring to the period directly preceding the reported current NA) to model CR effects from RU on the following NA. The within-level model is given by the following equations and graphically depicted in Appendix C:

13 $${RU}_{it}^{w}= {\phi }_{11i}{RU}_{i\left(t-1\right)}^{w}+{\phi }_{12i}{NA}_{i\left(t-1\right)}^{w}+ {\zeta }_{RUit}$$

14 $${NA}_{it}^{w}= {\phi }_{21i}{RU}_{it}^{w}+{\phi }_{22i}{NA}_{i\left(t-1\right)}^{w}+ {\phi }_{2.12i}({RU}_{it}^{w}{*{NA}_{i\left(t-1\right)}^{w})+ \zeta }_{NAit}$$

The employed model is a single-indicator model with all parameters estimated as random effects. Items were averaged per construct to create a single indicator variable. Note that this approach is not generally recommended but was chosen here to keep the example concise for illustrative purposes and because the available items were not well suited for a common factor model. Parameter estimates along with 95% CIs for all model parameters are provided in Tables A5 and A6 in Appendix C. CR and moderation effects were within-person standardized (std.) and subsequently averaged over persons. The results support the notion of mutually and self-reinforcing dynamics of NA and RU. That is, on average, next to positive AR effects, RU is associated with subsequent higher NA and vice versa (${RU}_{t}$ → ${NA}_{t}$; std. ${\gamma }_{{\phi }_{21}}=0.279$; and std. ${NA}_{(t-1)}$ → ${RU}_{t}$; ${\gamma }_{{\phi }_{12}}=0.126$). Furthermore, NA and RU show a positive interaction effect on subsequent NA; that is, on average, they have a reinforcing effect on subsequent NA (${RU}_{t}*{NA}_{(t-1)}$ → ${NA}_{t}$; std. ${\gamma }_{{\phi }_{2.12}}=0.048$). All of the AR and CR effects as well as the interaction effect show substantial inter-individual differences. For instance, the central 95% of individual (standardized) interaction effects lie in the interval [− 0.092; 0.210]. That is, while most individuals’ moderation parameters are positive, this is not the case for all individuals, suggesting that the reinforcing dynamic does not universally apply to all individuals. To better capture the amount of inter-individual differences in the dynamics, Fig. 3 illustrates the distribution of person-specific (standardized) AR, CR, and moderation effects for the outcome NA, and their associations across persons. The figure shows that there is a small tendency for individuals with higher AR in NA to have a smaller CR effect of RU on subsequent NA (random-effect correlation of the unstandardized effects $r({\phi }_{22},{\phi }_{21})=-0.295$), while the size of the individual moderation effect is not associated with the size of the AR or CR effect. Further random-effect correlations indicate that individuals with higher trait NA or RU tend to have RU and NA dynamics with higher innovation variances. For a complete list of between-level random-effect correlations see Table A6 in Appendix C.

### Discussion of the empirical examples

The present reanalysis of existing datasets by Blanke et al. (2020) and Houben et al. (2023) serve to illustrate the possibilities of multilevel latent moderated VAR models and replicate results previously reported on the within-person dynamics of NA, RU, and MF. In line with Selby et al. (2016), we observed synergistic, mutually reinforcing effects of RU and NA on subsequent NA. In line with Blanke et al., (2020; using the same data) but contrary to the findings by Bolzenkötter et al. (2024), we observed that MF weakens the effect of RU on subsequent NA. Please note that, as illustrative examples, the reanalyses do not aim to provide a comprehensive investigation of the respective substantive research questions. Naturally, the present illustrative examples have several limitations. First, with respect to methodological issues, we did not account for non-equidistant time intervals between measurements (across time and individuals). AR and CR effects do, however, substantially depend on the time interval between consecutive observations, and it is strongly recommended to account for varying lengths of time intervals by an adequate modeling strategy. The most straightforward way to include a proper handling of non-equidistant intervals in the model implementations provided is by inserting missing values based on a previously defined time grid (see Appendix A in Asparouhov et al., 2018). As described above, the Stan model codes provided for this application already include a respective missing data handling process; however, the preparation of the dataset and missing indicators has to be done by the researcher and depends on the time stamp information included in the respective datasets.

Second, the relationship between NA, RU, and MF may vary substantially across persons (see for instance empirical example 2). Accordingly, Hamaker et al. (2018) found substantive random-effect variances in a multilevel VAR model of NA, and Wenzel et al. (2023) report substantive random-effect variances in the effects of MF on NA. Consequently, a random-effects model might be better suited for modeling the moderation of NA-RU dynamics by MF in empirical example 1. The respective dataset did, however, not meet the necessary requirements for a random-effects multilevel moderated latent VAR model that were derived in the simulation study.

Third, on the substantive side, we focused on mindful attention and rumination as emotion regulation strategies. However, it was pointed out that individuals tend to combine different emotion regulation strategies concurrently instead of employing a single strategy in isolation (e.g., Brans et al., 2013). Accordingly, Blanke et al. (2020) considered different MF (present-moment attention and nonjudgmental acceptance) and emotion regulation strategies (rumination and reflection), with differential effects on affect. Consequently, we cannot rule out the possibility that the observed effects are confounded with the effect of simultaneously employed additional strategies other than mindful attention. Wenzel et al. (2023) investigated and observed differential effects for mindful attention and reappraisal as regulation strategies and provide a detailed discussion of further relevant mechanisms and factors such as the costs associated with employing different regulation strategies.

Last but not least, the chosen model (e.g., lagged vs. simultaneous effects, number of interaction effects) should depend on the substantive research question and the hypothesized mechanisms behind the investigated dynamics. We regard the current data applications as useful illustrative examples, which do not meet the claim of employing the one best model for the current datasets.

## Simulation studies

The performance of the three within-level dynamic interaction models described above (Models A, B, and C in Fig. 1B) is investigated in several simulation studies. In the following, the extensions R for *random* and F for *factor* in the model names refer to models with random dynamic effects (AR, CR, interaction effects, and innovation (co)variances) across persons and the inclusion of a measurement model for the within-level latent factor, respectively. Note that the interaction effect in the single-indicator models is considered to be latent too, as the respective product term involves the within-level variables ${{\varvec{Y}}}_{it}^{w}$ which result from latent person-mean centering and are thereby not directly observable.

In a first step, in simulation study I, we investigated the effect of several design factors for Model A (see Eqs. 4 and 5) in the fixed-effects model variant only. Here we tested the effect of (1) possible collinearity between the variables caused by and associated with different values of the AR and CR parameters, and (2) the reliability of the indicators on estimation accuracy in Model A in both the single-indicator and multiple-indicator (factor model) variants. The models in simulation study I were tested in relatively small samples of $N=100$ with short time series of $T=50$. Note that the estimation of (multilevel) time-series models requires ILD, that is, comparatively large numbers of observed time points per person, with recommendations of *T* ≥ 50 or *T* ≥ 100 being common for complex *N* = 1 or multilevel random-effects time-series models (see, e.g., Asparouhov et al., 2018; Schultzberg & Muthén, 2018; Schuurman et al., 2015).

The aforementioned two design factors were investigated for the following reasons. In a recent paper, Ariens et al. (2023) illustrated that the covariance between time-varying variables in AR(1) models with a time-varying covariate (Model A in Fig. 1B without an interaction effect) tends to increase not only with the size of the CR effect but also with the serial dependence (autocorrelation) in the time-varying covariate, potentially resulting in issues of collinearity, reduced estimation precision, and, in extreme cases, interpretation difficulties due to trade-off behavior between correlated estimates. They showed that estimation precision varies as a function of the effect sizes (AR and CR) as well as the time-series length, with alleviating effects of longer time series. Collinearity might be aggravated by the inclusion of interaction effects between the time-varying variables; however, the effects of collinearity on estimation accuracy in interaction models tend to be complex, with potential benefits with respect to power for detecting the interaction effect (McClelland & Judd, 1993; McClelland et al., 2017; Shieh, 2010). To gauge the extent to which estimation performance in the present simulation depends on the simulated AR and CR values, we systematically varied the combination of the data-generating values of the fixed AR and CR parameters. Data-generating values for ${\phi }_{11}, {\phi }_{22},$ and ${\phi }_{21}$ were varied while (a) holding innovation variances constant or while (b) simultaneously adjusting innovation variances in Model A (fixed-effect, single-indicator model variant). In total, nine different sets of parameter values were tested, with the chosen values mimicking moderate to relatively large AR and CR effects. A complete list of the (standardized) data-generating values is provided in Table 1 in the OSM.

The reliabilities of the indicators were varied, as previous studies have reported an effect of reliability on the estimation accuracy of AR effects, with detrimental effects of low reliability in single-indicator models which do not account for measurement error (Du & Wang, 2018; Koslowski & Holtmann, in press; Schuurman & Hamaker, 2019; Schuurman et al., 2015; Staudenmayer & Buonaccorsi, 2005). We varied the indicator reliabilities for Model A (fixed-effects, single-indicator variant) and Model A factor (multiple-indicator variant) to test whether reliability also affects estimation accuracy in models that do account for measurement error in the time series. Measurement error variances were generated such that within-level item reliabilities were approximately .5, .72, and .9 (see Table 2 in the OSM).

**Table 2:** Convergence rates for the random-effects models in simulation study II

*N*	*T*	Strict convergence criteria				$\widehat{R}<1.05$only			
		A-R	A-RF	A-R inncov	A-RF inncov	A-R	A-RF	A-R inncov	A-RF inncov
100	50	0.00	-	0.00	-	48.67	-	68.00	-
	100	57.33	20.67	54.00	19.33	93.33	89.33	96.67	90.67
	150	99.33	86.67	95.33	70.67	100.00	98.00	100.00	100.00
	200	100.00	98.00	99.33	94.67	100.00	100.00	99.33	98.67
150	50	0.00	-	0.67	-	56.00	-	72.00	-
	100	94.00	51.33	78.67	34.00	98.67	96.00	99.33	95.33
	150	100.00	98.67	100.00	80.67	100.00	100.00	100.00	100.00
	200	100.00	100.00	100.00	98.00	100.00	100.00	100.00	100.00
200	50	4.00	-	3.33	-	54.00	-	73.33	-
	100	99.33	-	92.00	34.00	100.00	-	99.33	96.67
	150	100.00	-	100.00	86.67	100.00	-	100.00	100.00
	200	100.00	-	100.00	98.00	100.00	-	100.00	100.00

Convergence rates are given in % of converged replications. The strict convergence criterion corresponds to$\widehat{R}<1.01$, no divergent transitions, and bulk and tail effective samples sizes > 100 per chain per parameter. *T* = 50 was not estimated for the multiple-indicator factor model variants (*F*). *N* = between-level (person) sample size; *T* = number of time points per person

Simulation study II focuses on Model A and covers models of different complexity, comparing (1) fixed- and random-effects models with the within-person dynamic parameters assumed as either constant or varying across persons in the multilevel model (termed Model A and Model A-R), (2) single-indicator and multiple-indicator (factor) models (termed Model A and Model A-F), with the latter assuming and modeling measurement error in the within-level process via means of dynamic factor analyses (see Fig. 1A), (3) modeling innovation covariances versus assuming innovations to be independent (testing the effect of model complexity; indicated by the term *inncov* in the model name), and (4) combinations of the former (e.g., Model A-RF inncov). Each of the resulting models is investigated under different between-level (person; *N*) and within-level (time points; *T*) sample sizes. For the fixed-effects models, these were chosen as *N* = 25, 50, 100 and *T* = 25, 50, 100, 200, in a fully crossed design. For the random-effects models, these were chosen as *N* = 100, 150, 200 and *T* = 50, 100, 150, 200, in an (almost) fully crossed design (see Table 2). The chosen person sample sizes and time-series lengths align with previous simulation studies and recommendations for (multilevel) time-series models, with values for *T* that can be considered relatively short in terms of time-series models but which are still realistic for AA studies (Adolf et al., 2017; Jongerling et al., 2015; Schultzberg & Muthén, 2018).

In simulation studies III and IV, we investigate the performance of Models B (see Eqs. 7 and 8) and C (see Eqs. 9–11 and Fig. 1B), respectively, focusing on fixed-effects models in the multiple-indicator (factor) version, including innovation correlations (i.e., models B-F inncov and C-F inncov). To keep estimation times reasonable, the model variants (fixed vs. random, single- vs. multiple-indicator) tested in simulation study II were not fully crossed in simulation studies III and IV. Simulation studies III and IV thereby focus on the performance of different, more complex within-level dynamic latent interaction models under different sample sizes, with *N* = 25, 50, 100 and *T* = 25, 50, 100, 200, fully crossed.

For each of the four simulation studies, we provide a detailed description of each simulated model, including model figures, model equations, the respective Stan model codes, the data-generating values, and the simulation results in the OSM.

### Data generation

In each of the simulation conditions, we generated 150 datasets in R (R Core Team, 2019). Within-level time series were generated of length 500 + *T*, and the first 500 data points were subsequently discarded as burn-in. For random-effects models, we inspected all generated person-specific parameters to ensure that these fell within the permissible parameter space and checked the generated within-level time series with respect to stationarity. Between-level values that violated the above conditions were discarded and replaced.

Data-generating values for AR and CR effects and their random-effect variances were chosen to resemble values typically observed in (multilevel) VAR models and employed in previous simulation studies (e.g., Adolf et al., 2017; Jongerling et al., 2015; Ozkok et al., 2022; Schuurman & Hamaker, 2019). Furthermore, we considered restrictions on the magnitudes and combined values of the within-level AR and CR coefficients, innovation variances, and their respective random-effect variances to achieve local stationarity (e.g., AR effects in a range of −1 and 1 at each measurement occasion) of the individual time series. This procedure yielded the following parameter values. We generated the fixed effects of AR coefficients in the range $0.3 \le {\gamma }_{{\phi }_{qq}}\le 0.4$ and of CR coefficients in the range $0.1 \le |{\gamma }_{{\phi }_{qq{\prime}}}| \le 0.25$ (depending on the respective model). Ozkok et al. (2022) suggested within-level interaction effects of 0.3 as moderate values; however, previous results on moderation effects in time series models are sparse. The few exceptions include, for instance, Koval and Kuppens (2012) reporting an experimentally induced effect of social stress anticipation on emotional inertia (moderated AR) of − 0.11, and Adolf et al. (2017) reporting the AR and CR effects of NA and perceived stress being dichotomously moderated by the time-varying presence of negative events as varying between − 0.25 and 0.75 for AR and − 0.75 and 0.3 for the CR effects across persons, with a vast majority of the effects being closer to zero. To test the models under strict conditions and yield conservative recommendations for *N* and *T*, we chose to set the fixed effects of the interaction effects to rather small values, with $0.05 \le |{\gamma }_{{\phi }_{k.qq{\prime}}}|\le 0.20$. Note that due to the different number of interaction effects in the models, the simulation studies also differ in some of the data-generating values (see below). In the random-effects models, random-effect variances of the $\phi$ coefficients were set to 0.01 (Jongerling et al., 2015; Schuurman & Hamaker, 2019), and not modeled (equaling zero) in the fixed-effects models. The fixed effects of the log innovation variances were generated as ${\gamma }_{\sigma \delta q}=-0.5$ in models without and as ${\gamma }_{\sigma \delta q}=-0.7$ with ${\gamma }_{\sigma \eta \zeta }=-1.3$ in models with a random innovation covariance (see OSM for the respective model parameterization), with a random-effect variance of ${\tau }_{\sigma \delta q}^{2}={\tau }_{\sigma \eta \zeta }^{2}=$ 0.2. For the latent factor models, we generated factors based on three continuous indicator variables per factor, with factor loadings set to ${\lambda }_{W}={\lambda }_{B}=1$ and within-level measurement error variances ${\sigma }_{{\varepsilon }^{W}}^{2}=0.3$, yielding item reliabilities of .69 to .79 (mean = .72, median = .71). Three indicators per factor were chosen, as this resembles the most parsimonious design that generally allows for the free estimation of factor loadings, while more than three indicators are oftentimes not available in AA data, which are usually collected with a design that aims to keep participant burden minimal. Between-level error variances were set to zero and not included in the model estimation; between-level trait variables correlated to ${\rho }_{{\mu }_{1},{\mu }_{2}}=.3$. The remaining model parameters were chosen to yield intra-class correlations of 0.4–0.5, as often found in longitudinal studies, and to keep simulated data within a range of 0 to 10. Standardized fixed effects of the within-level process parameters used in the data-generating model were calculated based on the standardization procedure described in Appendix A.^4^ Detailed lists of data-generating values for all parameters in each of the models, including standardized AR, CR, and interaction effects, are provided in the tables in the OSM.

### Model estimation, prior distributions, and evaluation criteria

Models were estimated in the free software Stan (Carpenter et al., 2017) via the interface provided in the rstan-package (Stan Development Team, 2023). Stan model codes and accompanying R scripts are available on OSF. MCMC sampling was done using two MCMC chains with 4,000 iterations per chain, with 50% of the respective iterations used as burn-in (not included in the posterior distribution). Model convergence was assumed when, for all monitored parameters, (1) $\widehat{R}$ was below 1.01, indicating adequate chain mixing, (2) the minimum estimated bulk and tail effective sample sizes across parameters exceeded 200 (i.e., > 100 per chain), and (3) no divergent transitions emerged. Replications that failed to meet the cutoff criteria were rerun, using twice the number of initially chosen iterations, and ultimately excluded from further analyses without replacement if cutoff criteria were still not met.^5^ The following estimation performance metrics are considered: (1) 95% coverage rates (values > 91% are considered adequate), (2) relative bias (posterior mean divided by the respective data-generating parameter value), with a maximum bias of up to 10%, i.e., relative bias in the range 0.9 to 1.1 considered adequate, (3) the mean squared error (MSE), and (4) empirical power based on the 95% credibility intervals of parameter posterior distributions (for data-generating values different from zero), with a minimum of 80% power considered adequate.

(Hyper)priors on the parameters were selected to be weakly informative, considering (a) the data characteristics, for example, the possible range of the observed variables (that is, the range that can be reasonably expected given the response formats used to obtain the observed variables), (b) reasonable ranges within which factor loadings can be expected to fall for a well-fitting factor model (note that the first factor loading per factor is fixed to 1), and (c) theoretically admissible ranges of autoregressive and cross-lagged parameters in stationary time-series models (admissible-range-restricted priors; see McNeish, 2019). That is, priors are considered to contain little information on the parameter locations within the admissible and theoretically plausible parameter space of the respective parameter. The chosen prior distributions were ${\gamma }_{{\mu }_{q}}\sim N\left(0,{4}^{2}\right)$ and ${\gamma }_{\phi }\sim N(\text{0,1})$ for the fixed effects of the random intercepts and within-level regression coefficients, respectively, with ${\tau }_{{\mu }_{q}}\sim Cauchy(\text{0,1})$ and ${\tau }_{\phi }\sim Cauchy(0, 0.5)$ for the respective random-effect scales (the latter if applicable). Note that the between-level random-effect covariance matrix is modeled via the decomposition into a correlation matrix and its scales, with an LKJ(1) hyper-prior set on the Cholesky factor of the correlation matrix (see Stan model codes on OSF and the tutorial in Appendix D; for details on the LKJ prior for Cholesky factors see Stan Development Team, 2023). The same holds for the within-level covariance matrix of the innovations in the fixed-effect models, i.e., if innovation variances were modeled as fixed across persons, with ${\sigma }_{{\zeta }_{q}}\sim Cauchy(\text{0,1})$ and an LKJ(1) prior for the Cholesky factor of the correlation matrix. In random-effects models with person-specific innovation variances and covariances, a latent variable approach (Hamaker et al., 2018) was chosen. The approach is explained in Appendix A. In this case, the logarithms of the innovation variances ${\text{ln}(\sigma }_{\delta qi}^{2})$ and covariances $\text{ln}\left({\sigma }_{\eta \zeta i}^{2}\right)$ were modeled within the multivariate normal distribution of the between-level random effects (see above), with a prior on the fixed effects corresponding to ${\gamma }_{\sigma \delta q}\sim N\left(\text{0,1}\right)$ and ${\gamma }_{\sigma \eta \zeta } \sim N\left(\text{0,1}\right)$, and priors for the scales as in the fixed-effects model, i.e., ${\tau }_{\sigma \delta q}\sim Cauchy(\text{0,1})$ and ${\tau }_{\sigma \eta \zeta }\sim Cauchy(\text{0,1})$. For latent factor models including a measurement model, respective priors were chosen as ${\lambda }_{W}\sim N(1, {0.5})$, $\alpha \sim N(0,{10})$, and ${\sigma }_{\varepsilon }\sim Cauchy(\text{0,1})$. As data were simulated with observed values in a range of 0 to 10, selected (hyper)priors for the random intercepts were chosen accordingly (and might not transfer to data observed in highly different ranges, e.g., in a range from 20 to 80). Applied researchers aiming to implement the presented model codes could change the location and scales of the prior for ${\gamma }_{{\mu }_{q}}$ and ${\tau }_{{\mu }_{q}}$ or apply a simple scale retransformation to their data to avoid making adaptations to the model priors.

### Simulation results

Detailed results (model convergence, relative bias, coverage rates, MSE, and power) are given per condition and/or parameter type for every simulated model in the OSM.

#### **Simulation study I, effect of multicollinearity**

A comparison of the results for the different data-generating values for AR effects, CR effects, and innovation variances in Model A (fixed-effects model) shows that under *N* = 100 and *T* = 50, all parameters were accurately estimated (according to all performance criteria) irrespective of the data-generating values. Detailed results are presented in Sect. 2 of the OSM. Data-generating values of the within-level VAR model were therefore not further considered (that is, not further varied) in the following simulations.

#### **Simulation study I, effect of reliability**

**Fig. 4:**
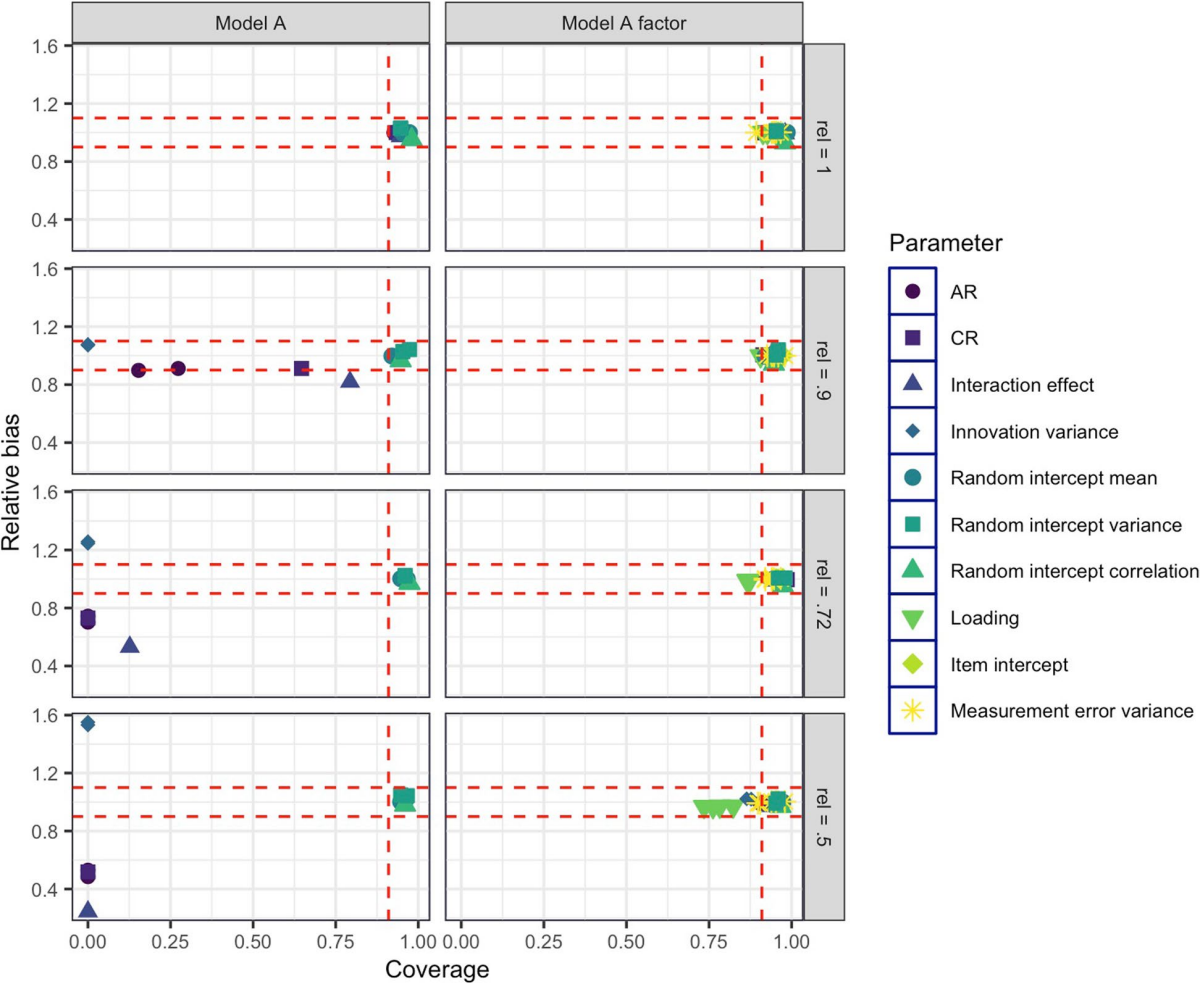
Results of simulation study I, reliability. Relative bias and coverage for Model A and Model A factor (including a measurement model) for different values of indicator reliability. Parameter types are coded by color and symbol. The red dotted lines depict the chosen cutoff values (relative bias < 10%, that is, relative bias between 0.9 and 1.1; coverage rates between .91 and .98). AR: autoregressive effect; CR: cross-regressive effect

Convergence statistics and values of all evaluation criteria are presented in Sect. 2 of the OSM. Relative bias and coverage values are depicted in Fig. 4. Model convergence was close to 100% under all generated conditions for both Model A and A-F. As expected from previous studies on the effect of measurement error in single-indicator time-series models, low reliability was associated with substantial bias and low coverage rates in AR, CR, and the moderation effect, as well as innovation variances in Model A. Relative bias stayed below the cutoff of 10% for all parameters except the moderation effect in the case of a reliability of .9 and exceeded the cutoff values for reliabilities of .72 and .5. Power for the moderation effect stayed high except for the lowest reliability of .5. The results support previous findings that indicate that measurement error in the observed time series may bias the estimation of AR (and associated) effects. These results suggest that not accounting for measurement error in time series of measures with less than perfect reliability is not recommended.

In Model A-F, which explicitly modeled measurement error by the inclusion of a measurement model, the indicators’ reliabilities only had negligible effects on the relative bias and power of all model parameters, with all values meeting the expected values for low bias and high power. Coverage values were good for all parameters with the exception of log innovation variances in the lowest reliability condition of .5 (coverage = .86–.87) and loading parameters ${{\varvec{\lambda}}}_{W}$ in conditions with reliabilities of .72 and .5. Based on these results, which suggest only minimal effects of reliability in multiple-indicator models, the indicators’ reliability is not further varied in the following simulation studies, but is fixed to medium-sized values (on average .72, median reliability = .71).

#### **Simulation study II, fixed-effects models**

**Fig. 5:**
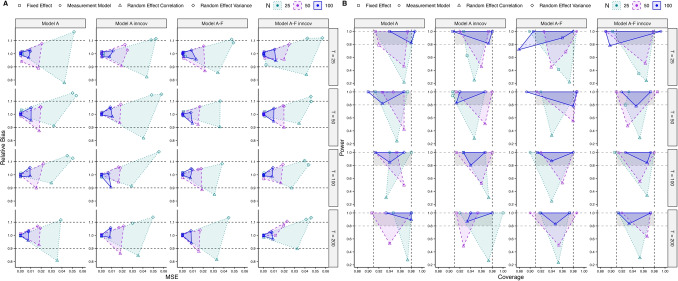
Results of simulation study II, fixed-effects models. **A** Relative bias and mean squared error (MSE). **B** Power and 95% coverage. Depicted is the convex hull spanned by the most extreme values observed for the parameter estimates on the respective two performance criteria. Parameter types are plotted by different symbols, indicating whether any parameter type consistently accounted for the extreme (border) values in the convex hull. Sample sizes are coded by color and line type. The gray dotted lines depict the chosen cutoff values (relative bias < 10%, that is, relative bias between 0.9 and 1.1; coverage rates between .91 and .98; power > .8). F in the model names indicates dynamic factor models (including a measurement model); Model A/A inncov: model without/with innovation covariance

Convergence rates for the fixed-effects models were > 84% across conditions, with the lowest values in the latent model variants with *T* = 25 (convergence being > 96% for *T* ≥ 50). Results for the performance criteria for the fixed-effects models of simulation study II are depicted in Fig. 5. Plots show the convex hull spanned by the different parameters’ respective values, per simulation condition. That is, the depicted shaded hulls (shaded areas) cover the values of the respective criteria of all of the parameters in one condition, with the most extreme values defining the points that span the lines of the hull. These most extreme values of bias/MSE/power/coverage in a specific condition thereby build the edges of the shaded areas, with the symbols representing the parameter type that assumes this value. Parameters that are not explicitly depicted lie within the shaded areas. If the entire shaded area lies within the area of the chosen cutoff values (e.g., relative bias < 10%, that is, relative bias between 0.9 and 1.1), this shows that none of the parameters exceeds this cutoff. Additionally, all evaluation criteria (relative bias, coverage, MSE, power) are listed for every single parameter per condition in the tables and depicted in alternative figures in Sects. 3 and 6 in the OSM.

Across models and sample sizes *N* and *T*, it is apparent that the parameters defining the extremes are the between-level random-effect (co)variances (i.e., in these models these are only the individual-specific traits/latent averages). Considering only the within-level VAR model parameters, we can derive that Model A with fixed VAR parameters can already be accurately estimated with as few as *T* = 25 and *N* = 25, with either *T* or* N* ≥ 50 needed for sufficient power for the interaction effect. With respect to the between-level (co)variances, *N* ≥ 50 is recommended for accurate estimation and *N* ≥ 100 for sufficient power of the covariance/correlation. These recommendations hold for both the models without and with a measurement model (Model A and Model A-F), with similar results for both types of models. Parameters of the measurement models are estimated accurately across all simulated conditions as judged by all performance criteria.

#### **Simulation study II, random-effects models**

Convergence rates for the random-effects models are provided in Table 2. Detailed warnings and non-convergence reasons are listed in the tables in the OSM. Applying a strict convergence criterion as described above, convergence rates for Models A-R and A-R inncov (single-indicator models) suggest that *T* = 50 is not sufficient for this kind of model complexity, with convergence rates of 0%. For this reason, we no longer include the condition *T* = 50 in the respective more complex multiple-indicator (factor) model variants. Convergence rates in the respective conditions rise to 48.67–73.33% using a convergence criterion of $\widehat{R}<1.05$ per parameter, not considering divergent transitions or effective sample sizes. For *T* = 100, convergence rates depend on the between-level sample size and the specific model, with higher *N* needed for multiple-indicator factor models. For *T* ≥ 150, convergence is acceptable to very good across all *N*.

In the non-converged small *T* conditions, we investigated model convergence criteria separately for each parameter (detailed results are provided in the tables in Sect. 7 in the OSM). This revealed that non-convergence due to a strict convergence criterion was primarily driven by the random-effect variances of the AR, CR, and interaction effects. Further examining model convergence of these parameters showed that non-convergence was associated with low posterior means (tending towards zero) of the respective variance parameters as compared to converged replications of the respective condition, indicating that convergence issues are related to estimating random-effect variances in the case of only small inter-individual differences in the respective parameters. Using a convergence criterion of $\widehat{R}<1.05$ only, percentages of parameters with $\widehat{R}>1.05$ were almost equally distributed across parameter types, with slightly higher values for random-effect variances. These results indicate that divergent transitions and low ESS were mostly associated with small random-effect variances.

**Fig. 6:**
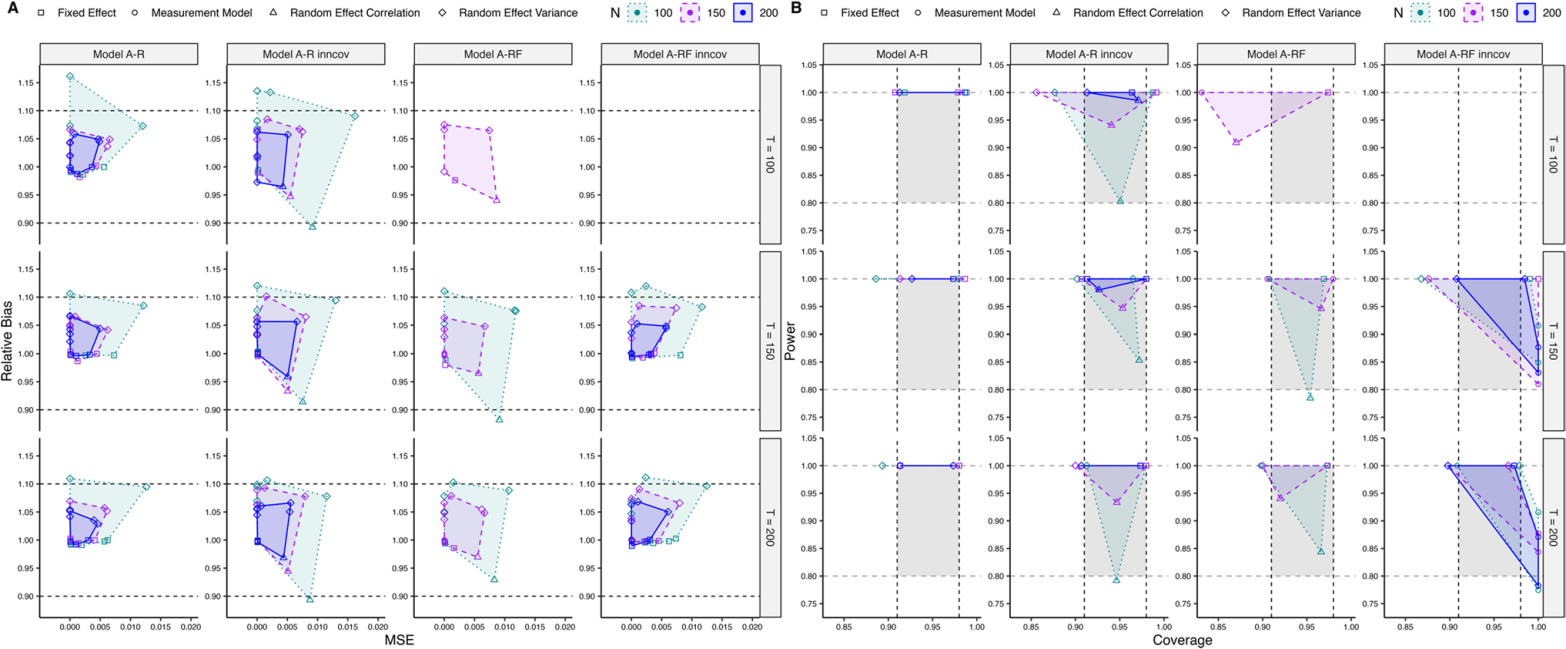
Results of simulation study II, random-effects models. **A** Relative bias and mean squared error (MSE). **B** Power and 95% coverage. Depicted is the convex hull spanned by the most extreme values observed for the parameter estimates on the respective two performance criteria. Parameter types are plotted by different symbols, indicating whether any parameter type consistently accounted for the extreme (border) values in the convex hull. Sample sizes are coded by color and line type. The gray dotted lines depict the chosen cutoff values (relative bias < 10%, that is, relative bias between 0.9 and 1.1; coverage rates between .91 and .98; power > .8). F in the model names indicates dynamic factor models (including a measurement model); Model A/A inncov: model without/with innovation covariance. Note that conditions with less than 50% converged replications are not depicted. Parameters with data-generating values equal to zero are not depicted (see respective tables)

Performance criteria for the random-effects models are depicted in Fig. 6. These are calculated using replications classified as converged based on the strict convergence criterion. Conditions with less than 50% converged replications according to this criterion are not depicted. Detailed values for all performance criteria under both convergence criteria (strict or $\widehat{R}<1.05$ only) are provided in the OSM. In the single-indicator models (A-R and A-R inncov), bias, coverage, MSE, and power are satisfactory for almost all parameters in all of the converged conditions (even under *T* = 100 with *N* = 100), with very small biases for a few of the random-effect variances of the $\phi$ parameters under *N* = 100 when using the strict but not when using the lenient convergence criterion. Using $\widehat{R}<1.05$ reveals that for *T* = 50, either coverage or relative bias values are slightly too low for a few of the random-effect variances (primarily the CR effect).

In the factor model variants (A-RF and A-RF inncov), *N* ≥ 100 with *T* ≥ 150 or N ≥ 150 with *T* ≥ 100 are sufficient for unbiased AR, CR, and innovation covariance random effects. Note that this is also the case for the *T* = 100 with *N* = 100 condition in the factor models (see OSM), which exhibited a large number of convergence issues under the strict convergence criterion, which largely disappeared using $\widehat{R}<1.05$ only. MSE is very small and power is satisfactory for all parameters in the mentioned conditions. Note that coverage values are generally acceptable to very good, however, with few irregular patterns across sample size conditions. We assume that this is most probably not due to the respective samples sizes *N* and *T*, but that using less than 150 replications, which resulted from convergence rates smaller than 100%, might not have been enough for a very precise estimation of coverage rates.

#### **Simulation study III**

**Fig. 7:**
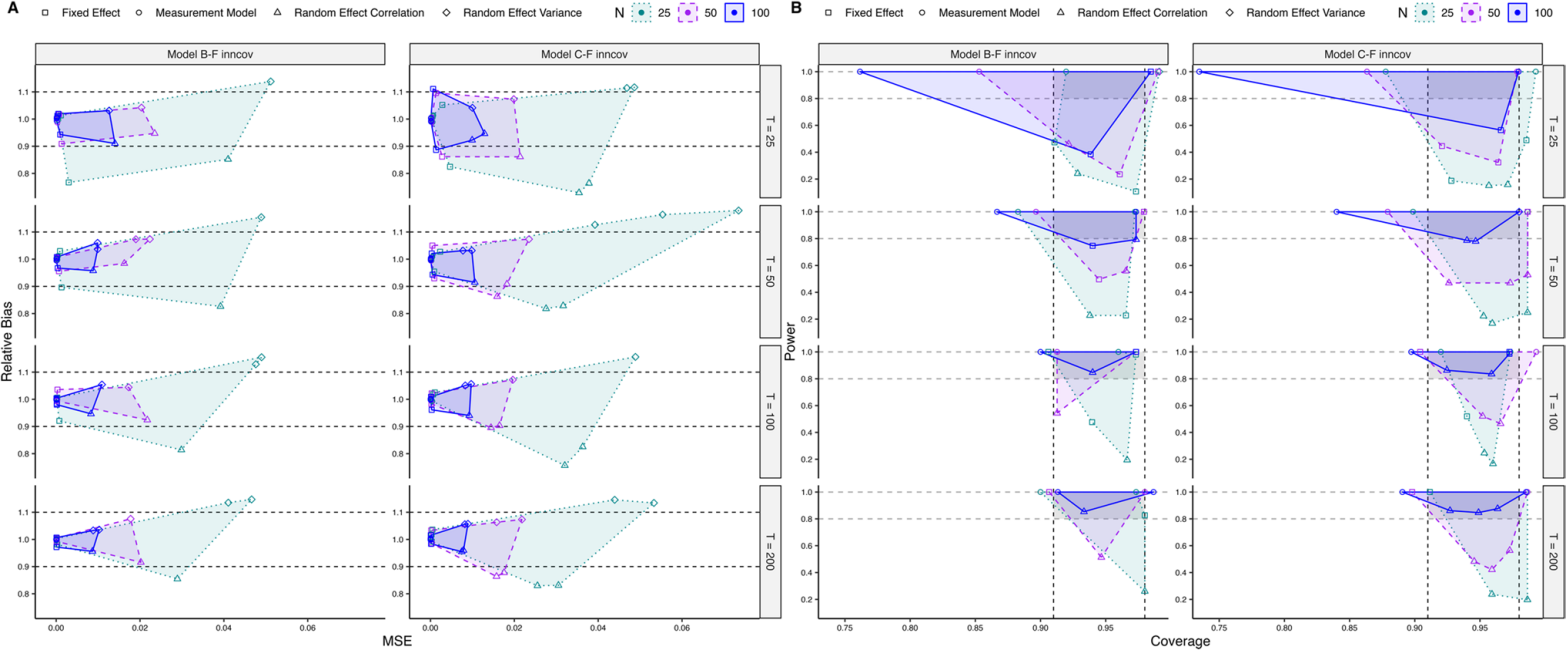
Results of simulation studies III and IV. **A** Relative bias and mean squared error (MSE). **B** Power and 95% coverage. Depicted is the convex hull spanned by the most extreme values observed for the parameter estimates on the respective two performance criteria. Parameter types are plotted by different symbols, indicating whether any parameter type consistently accounted for the extreme (border) values in the convex hull. Sample sizes are coded by color and line type

Convergence rates were > 68% in conditions with *T* = 25, and > 96% otherwise. Results for the performance criteria for Model B-F inncov (factor, fixed effects) are depicted in Fig. 7. Considering the within-level VAR model parameters, coverage rates and relative bias are within the desired limits for all parameters in all conditions, except for the relative bias of the interaction effect ${\phi }_{1.12}$ in the *T* = 25 with *N* = 25 condition. The latter bias disappears with either *T* or *N* ≥ 50. To achieve sufficient power for the interaction effects, either *N* ≥ 50 with *T* ≥ 100 or *T* ≥ 50 with *N* ≥ 100 are needed. Note that this difference from the respective model variant A (Model A-F inncov) is driven solely by the coefficient ${\phi }_{1.12}$, which is not included in Model A-F inncov. This interaction effect was generated with a smaller effect size as compared to the interaction effect ${\phi }_{2.12}$, which is a straightforward explanation for the smaller power to detect ${\phi }_{1.12}$ as compared to ${\phi }_{2.12}$. It appears that differences between Model A and B in the current simulation design are completely driven by this difference in effect size. With respect to the between-level (co)variances of the latent trait variables, again, *N* ≥ 50 is recommended in terms of bias, and *N* ≥ 100 for sufficient power of the covariance/correlation. Parameters of the measurement model are estimated without bias (rel. bias between 0.97 and 1.01; MSE < 0.002), with some coverage rates being slightly too low (> .76).

#### **Simulation study IV**

Convergence rates for Model C-F inncov (factor, fixed effects) were > 91% across all conditions. Results for the performance criteria are depicted in Fig. 7. Again, parameters of the measurement model are estimated accurately, with results similar to those in simulation study II. With respect to the within-level VAR model parameters, *N* ≥ 50 with *T* ≥ 50 is needed for accurate parameter estimation (relative bias), with good coverage values in all conditions. Power to detect the CR effects varied with the standardized effect sizes of the CR effect, with required sample sizes to reach a power > 80% being *N* ≥ 50 or, for *N* = 25, *T* ≥ 50 for the standardized parameters of $\left|\phi_{qq'}\right|=\;0.09-0.19$. For $\left|{\phi }_{q{q}{\prime}}\right|=0.05$ (standardized), the respective sample sizes are *N* = 25 with *T* ≥ 200, *N* = 50 with *T* ≥ 100, and *N* = 100 with *T* ≥ 50. Similarly, for the interaction effects, for the larger standardized effect of $\left|{\phi }_{k.q{q}{\prime}}\right|=0.12,$ adequate power requires *N* ≥ 50 or, in the case of *N* = 25, *T* ≥ 50. In comparison, the smaller standardized effect of $\left|{\phi }_{k.q{q}{\prime}}\right|=0.08$ requires *T* ≥ 100 for *N* = 25 and *T* ≥ 50 for *N* ≥ 50. With respect to between-level random-effect (co)variances, *N* ≥ 50 with *T* ≥ 50 or *N* ≥ 100 with* T* ≥ 25 are needed for precise estimation and *N* ≥ 100 with* T* ≥ 50 for adequate power of the covariances/correlations.

## Discussion of the simulation studies

The present simulation studies show that multilevel latent VAR models with within-level latent interaction effects can be estimated with reasonable sample sizes for models of this complexity. Based on the simulation results, the following recommendations can be derived. If researchers are primarily interested in the within-level dynamics and parameters can reasonably be assumed to be invariant across persons, i.e., persons share the same dynamics, we recommend *N* ≥ 50 with *T* ≥ 50. Note that this recommendation depends on model complexity and the effect size of the CR and interaction effects, with *T* ≥ 25 being sufficient for at least medium-sized effects in fixed-effects models. Sample sizes should be increased to either *N* ≥ 100 or *T* ≥ 100 if the CR effects or moderation effects are assumed to be (very) small. If researchers are additionally interested in the between-level covariances (of the latent trait variables), the number of persons should be increased to *N* ≥ 100. In cases where parameters are likely to vary across persons and a random-effects model is indicated, sample sizes should be increased to *T* ≥ 100 with *N* ≥ 100 or ideally *N* ≥ 150 in the single-indicator model. For random-effects factor models including a measurement model, *N* ≥ 100 with *T* ≥ 100 might be sufficient but could lead to convergence issues in the case of small random-effect variances. Alternatively, *N* ≥ 100 with *T* ≥ 150 or *N* ≥ 150 with *T* ≥ 100 should be included in the model.

Note that sample size recommendations depend on specific model characteristics (e.g., inclusion of a measurement model, number of random effects, between-level structural model) and are therefore not directly comparable with those of previous simulation studies on different (multilevel) (V)AR models. Our results are generally in line with previous results suggesting small sample size bias for random-effect variances (Andriamiarana et al., 2023; McNeish, 2016, 2019; Schultzberg & Muthén, 2018) and compensating effects between *N* and *T* (Andriamiarana et al., 2023; Schultzberg & Muthén, 2018). However, especially in the most complex random-effects latent moderated VAR models, a sufficiently large *T* is needed as a prerequisite for precise model estimation, with large *N* being able to compensate for somewhat smaller *T* only after this minimum threshold is surpassed.

In contrast to previous studies focusing on small sample behavior of Bayesian MCMC sampling in multilevel models (McNeish, 2019; Schultzberg & Muthén, 2018), we did not put the random-effects moderated VAR models to a genuine small sample test under different prior distributions. First, the effect of different prior settings and resulting small sample behavior was not the focus of the present study. Second, the fixed-effects models already suggested that bias and power of the random mean variance parameters require *N* ≥ 50 and *N* ≥ 100, respectively. This result is in line with the simulation study by Schultzberg and Muthén (2018) on single-indicator multilevel AR(1) models as well as with Andriamiarana et al. (2023). Results by Schultzberg and Muthén (2018) further demonstrated that modeling between-level associations of random means requires smaller sample sizes than those of random AR or innovation variances. Hence, it was to be expected that including random effects for all dynamic VAR parameters at the within-level would further increase the sample size demands above those observed for our fixed-effects models with random means.

The present simulation study is, naturally, limited with respect to the types of within-level VAR models that are covered as well as with respect to the simulated data characteristics, that is, the respective effect sizes of the parameters that drive the within-level dynamics. Based on the results of varying the data-generating values of the AR and CR effects, we are optimistic that the models can still be estimated well in case of larger effect sizes. However, researchers should be aware of potential collinearity issues that might arise due to time-varying covariates with (very) large effects on the dependent variable and a high autocorrelation in VAR models (see Ariens et al., 2023). Given the present results and sizes of interaction effects that can be reasonably expected in behavioral research, we consider small effect sizes to be a potentially larger problem in practice. That is, in the case of effects even smaller than those chosen for the present study, larger sample sizes might be needed to detect these effects. The chosen effect sizes for CR and moderation effects, can, however, be considered small as compared to values used in previous studies (see, e.g., Ariens et al., 2023; Ozkok et al., 2022).

Regarding model complexity, we did not consider different between-level structural models in the present simulation study. Sample size recommendations for different between-level latent regression models of within-level AR(1) model parameters are derived in Schultzberg and Muthén (2018). Andriamiarana et al. (2023) present a simulation study for a nonlinear dynamic latent class SEM including a between-level latent interaction effect. Furthermore, note that between-within-level interaction effects, as for instance discussed in Ozkok et al. (2022), are easily integrated into multilevel VAR models by relating the respective random within-level coefficients to variables on the between-level. These kinds of models are covered by the dynamic SEM framework (see Asparouhov et al., 2018; Hamaker et al., 2018; Schultzberg & Muthén, 2018). Researchers should keep in mind that, for similar between-level structural models, required sample sizes for multilevel moderated VAR models may differ from those in Schultzberg and Muthén (2018) due to the more complex within-level structural model and in the case that the between-level structural model includes random CR and interaction effects. In these cases, results regarding the respective random variances in the present simulation should be considered.

The sample size recommendations derived above are in many cases driven by the bias of between-level random-effect variances or the power of the random-effect covariances and correlations. Note that we used an LKJ(1) prior on the between-level correlations, which is uniform over the space of correlation matrices of the same size (see Stan Development Team, 2023), resulting in the marginal distributions of the correlations being increasingly concentrated over zero as the size of the matrix increased. With respect to random-effect variances, the use of diffuse priors has been shown to be prone to bias in small samples, emphasizing the importance of prior selection for between-level parameters (e.g., Andriamiarana et al., 2023; Depaoli & Clifton, 2015; McNeish, 2016, 2019; Schultzberg & Muthén, 2018; Schuurman et al., 2016b). Additionally, researchers should be aware that small variances of the between-level random effects might potentially hinder model convergence. Unfortunately, in multilevel VAR models, small between-level variances are omnipresent as the variances of the $\phi$ coefficients are bounded in stationary models (i.e., individual $\phi$ parameters have to stay within limits of coefficients that comply with stationarity of the individual time series). Convergence problems due to sampling random effects with small variances is a known problem that might be solved by use of an efficient model reparameterization, referred to as the non-centered parameterization (Papaspiliopoulos et al., 2007). Furthermore, estimation precision of the random-effect variances may depend on the choice of the priors. We strongly recommend testing the effect of different prior settings (within the realm of weakly informative admissible-range restricted priors) within a sensitivity analysis in every data application. Due to the separation of the covariance matrix into its scales and a correlation matrix, priors on random variance parameters can be easily adapted. A large variety of admissible-range restricted prior distributions are available that could be chosen. See, for instance, McNeish (2019) for a procedure on how to select admissible range-restricted prior distributions in multilevel AR(1) models. Schuurman et al. (2016b) discuss the use of data-based prior specifications for variance parameters. Note that we did not set informative priors centered over data-generating values or vary the prior specifications in any of the simulation conditions. As priors may have a non-negligible impact on sample size requirements, future research should focus on a thorough investigation of prior settings for the models presented.

That said, very small variances of random effects might also indicate that the respective random-effect variance is not substantial and that it might be converted to a fixed effect. Alternatively, as a first step, models may be simplified by excluding random-effect correlations of the respective effect from the model (Asparouhov & Muthén, 2022). Asparouhov and Muthén (2022) provide a discussion of several reasons for convergence issues in multilevel VAR models with accompanying recommendations.

## General discussion and conclusion

In the present article we discussed multilevel latent moderated VAR models for the analysis of latent interaction effects at the dynamic within-person level in ILD. The availability of ILD in the behavioral sciences has opened the opportunity to investigate increasingly complex questions regarding the joint dynamics of different variables that happen within the individual across time. Many research questions in the behavioral sciences are concerned with within-person dynamics and include hypotheses involving nonlinear effects. The presented multilevel latent moderated VAR models offer the possibility to model nonlinear longitudinal relationships as well as intra-individual changes in dynamics over time that are induced by time-varying covariates (e.g., situations, contextual information), while using latent person-mean centering and appropriately accounting for measurement error. Controlling for measurement error is essential in the behavioral sciences, where observations are prone to be measured with error, and in time series, where the estimation of the dynamic parameters are affected by measurement error in the series (e.g., Du & Wang, 2018; Schuurman et al., 2015). With the models presented herein, we build on previous work (Adolf et al., 2017; Kelava & Brandt, 2019) and provide a tutorial, a practical illustration, and a simulation study for multilevel latent VAR models.

While we focused on within-level interaction effects, the models can be easily extended to include both between-within-level and between-between level interactions between latent variables (see, e.g., Asparouhov et al., 2018; Asparouhov & Muthén, 2021). For instance, researchers might be interested in the joint effect of momentary self-esteem and tense arousal on self-injurious urges in patients with borderline personality disorder (e.g., Santangelo et al., 2020). The presented models could incorporate a moderating effect of momentary self-esteem as well as of trait self-esteem on the dynamic relationship between NA and self-injurious urges.

The presented models can accommodate both latent continuous moderator variables and observed categorical moderator variables for the dynamics of latent factors across time. If researchers assume that changes in the variables’ dynamics over time are rather due to unobserved entities and wish to model unobserved heterogeneity in the intra-individual trajectories, we recommend approaches combining multilevel latent VAR models with a hidden Markov model process, that is, DLCA (Asparouhov & Muthén, 2017) or the NDLC-SEM approach by Kelava and Brandt (2019). In these approaches, individuals can enter different states across time, with the states defining the respective within-state dynamics and time-dependent state membership following a Markov process with individual-specific transition probabilities.

We discussed basic variants of within-level interactions in the multilevel moderated VAR models in this article, which might be adapted to different research questions, for instance by an extension to more than three constructs, different types of interaction effects, or AR and CR effects of order larger than 1. However, researchers should keep in mind that model estimation tends to become more difficult with increasing model complexity, due to the increased number of effects that are to be estimated and potential challenges due to small effect sizes or close to zero random-effect variances. In general, to avoid overfitting, we advise researchers to keep their models as parsimonious as possible given the substantive research question at hand. We therefore recommend using theoretical reasoning to determine how many and which interaction effects to include in a model. Additionally, competing models should be compared based on model fit statistics. At this moment, we have not yet integrated the calculation of fit diagnostics into the model implementations. There are several options for Bayesian model evaluations that might be of interest, such as graphical posterior predictive checks or the model’s future predictive accuracy via leave-one-out cross-validation or the widely applicable information criterion (WAIC) (Bürkner et al., 2020; Vehtari et al., 2017). Future work should focus on the integration of model evaluation diagnostics into the presented models.

Furthermore, additional challenges for the models’ application in practice may arise due to a nesting of observations in days with a respective need to control for overnight lags or varying time intervals between observations. In the empirical example, we show one option for dealing with a nesting in days which ensures that overnight lags are not included in the estimation of the AR and CR effects. However, there are other possibilities to deal with overnight lags, e.g., by treating the night as a series of missing data points (possibly in combination with accounting for varying time intervals; see Asparouhov et al., 2018).

The models were implemented using Bayesian MCMC sampling in the Stan software program, which offers great flexibility of model specification and allows researchers to implement and adapt models to their needs, including complex nonlinear relationships between variables, latent variable models, and multilevel time-series models. However, the Bayesian estimation approach entails the challenge of choosing appropriate prior distributions for the model’s parameters. Prior distributions may have a substantial impact on convergence and estimation accuracy (e.g., Depaoli & Clifton, 2015; McNeish, 2016, 2019; Schuurman et al., 2016b), especially in small samples, and should therefore be chosen cautiously. We opted for the use of weakly informative admissible-range-restricted priors on most of the model parameters, which do not rely on previous studies’ results and were shown to improve estimation of multilevel time series in small samples (McNeish, 2019). More informative priors, which might be based on prior information retrieved from the literature, might improve convergence and reduce sample size requirements but should be chosen with great care, due to potential detrimental effects in case of inaccurate prior locations (Depaoli, 2014; Holtmann et al., 2016).

The recommended sample sizes vary from *N* ≥ 50 with *T* ≥ 25 in the simplest fixed-effects model with at least medium effect sizes for CR and interaction effects, up to *N* ≥ 100 or ideally *N* ≥ 150 with *T* ≥ 100 in random-effects factor models. Large *N* can only partly compensate for the size of *T* in random-effects models, as a sufficiently large *T* is paramount for estimating person-specific within-level dynamic parameters. Note that the recommended sample sizes for *T* are not unusual for random-effects (or *N* = 1) time-series models of this complexity (see, e.g., Asparouhov et al., 2018; Schultzberg & Muthén, 2018; Schuurman et al., 2015).

In summary, in this article we illustrated how multilevel latent moderated VAR models can be used to model latent interaction effects at the dynamic within-person level and provided guidance on the applicability of different model variants under different sample sizes. The presented models allow researchers to investigate research questions regarding complex relationships between variables across time while accounting for measurement error in the observed variables. We make the model implementations available and the model codes more accessible to applied researchers by providing both the Stan model codes of the presented models, which can be adapted by applied researchers to their respective needs, and a tutorial which explains the implementation of a fixed- and a random-effects model variant, illustrating the different model components. The goal of these commented code versions is not to provide an introduction to the Stan modeling language or Bayesian estimation methods in general. However, we do hope that in combination with respective introductions (see, e.g., Li et al., 2021, for a tutorial on fitting basic multilevel VAR models in Stan; Sorensen et al., 2016, for a tutorial on fitting hierarchical models in Stan; and van de Schoot et al., 2014, for an introduction to Bayesian analysis), the provided tutorial will make multilevel latent moderated VAR models accessible to a wide range of researchers.

## Supplementary Information

Below is the link to the electronic supplementary material.

Supplementary file1 (PDF 636 KB)

## Data Availability

The reanalyzed datasets were taken from https://osf.io/nvt6a/ and https://osf.io/vt2q5/

## Footnotes

1 Note that for reasons of comparability, we detrended all observed variables based on the measurement–occasion counting variable, analogous to the procedure in Blanke et al. (2020).

2 To stay in line with the original analyses, we did not further account for different time intervals between observations. Note, however, that unequal time intervals between observations could be easily handled within the proposed models by using an approximate approach as proposed by Asparouhov et al. (2018). The approach is based on inserting missing values to approximate equidistant time intervals based on a user-specified time grid, which are subsequently sampled alongside the remaining model parameters within the MCMC sampling. In addition to this approximate approach, exact continuous time dynamic models are available (e.g., Driver & Voelkle, 2018; Voelkle et al., 2012). An extension of the presented models to continuous time models is, however, beyond the scope of the present article.

3 Note, however, that the absolute values are not directly comparable, as Blanke et al. (2020) report unstandardized effects only, and the model slightly differs with respect to the inclusion of nonjudgmental acceptance as a second MF facet.

4 Due to a lack of an analytical solution for the latent within-level variables’ standard deviations in time series models with interaction effects, we calculated the population values of these standard deviations by a simulation procedure. That is, we generated a time series of length 10^6^ and calculated the standard deviation for this time series.

5 Following the suggestion of an anonymous reviewer, we recalculated convergence rates and evaluation criteria in the random-effects models for the common and less strict criterion of $\widehat{R}<1.05$ only. See results section.

## Code availability

All model and analysis codes for this study are provided on the Open Science Framework and can be accessed via https://osf.io/bvsqy

## Declarations

### Open practices statement

All materials (model and analyses codes) for this study are provided on the Open Science Framework and can be accessed via https://osf.io/bvsqy

Computations for this work were done (in part) using resources of the Leipzig University Computing Centre.
