# Supplementary material for: Modeling within-level latent interaction effects in multilevel vector-autoregressive models
Source: Behav Res Methods. 2025 Sep 5;57(10):277. doi: 10.3758/s13428-025-02694-3 (PMC12413427; doi:10.3758/s13428-025-02694-3)
Supplement: Supplementary file 1 — Supplementary file1 (PDF 636 KB) [file 13428_2025_2694_MOESM1_ESM.pdf]

Appendix to:

Modeling within-level latent interaction effects in multilevel vector-autoregressive models

Jana Holtmann & Kenneth Koslowski
Leipzig University

Appendix A

Supplementary descriptions of modeling strategies

A.1 Modeling random (person-specific) innovation covariances

In models which assume random innovation (co)variances across persons, which receive a distribution across persons with a respective average level (fixed effect) and (random effect) variance, the implementation of the model via a multivariate normal distribution at the within-person level is not the most efficient.

We therefore follow an approach suggested by Hamaker et al. (2018), modeling the person-specific innovation covariances by introducing a new, common factor $\eta_{\zeta it}$. That is, the innovations ζ_{1it} and ζ_{2it} are split into two components, a factor common to both innovations, which captures the shared part of the fluctuations, and a respective residual term δ_{kit} which is unique to construct k:

$$\zeta_{1it} = \eta_{\zeta it} + \delta_{1it}, \qquad \zeta_{2it} = \eta_{\zeta it} + \delta_{2it}$$

The factor $\eta_{\zeta it}$ captures fluctuations that are common to both constructs, while ζ_{1it} and ζ_{2it} capture unique, construct-specific, uncorrelated components of the innovations, which are assumed to be iid for each person i, with

$$\eta_{\zeta it} \sim N(0, \sigma^2_{\eta_\zeta i}), \quad \delta_{1it} \sim N(0, \sigma^2_{\delta_1 i}), \quad \delta_{2it} \sim N(0, \sigma^2_{\delta_2 i})$$

The sign of the innovations' covariance has to be a priori specified as either positive or negative for all individuals (Hamaker et al., 2018), achieving a negative correlation by fixing the factor loading of $\eta_{\zeta it}$ to minus one for either $k = 1$ or $k = 2$ in

$$\zeta_{kit} = \eta_{\zeta it} + \delta_{kit}$$

. Innovation (co)variances are obtained by

$$\sigma_{\zeta_{12} i} = \sigma^2_{\eta_\zeta i}, \quad \sigma^2_{\zeta_1 i} = \sigma^2_{\eta_\zeta i} + \sigma^2_{\delta_1 i}, \quad \sigma^2_{\zeta_2 i} = \sigma^2_{\eta_\zeta i} + \sigma^2_{\delta_2 i}$$

This approach ensures positive definite innovation covariance matrices per individual (Hamaker et al., 2018) and allows for the joint modeling of innovation covariances' and residual variances' logarithms ($ln(\sigma^2_{\eta_\zeta i})$, $ln(\sigma^2_{\delta_1 i})$, and $ln(\sigma^2_{\delta_2 i})$) with the remaining random effect parameters on the between-person level. Innovation covariances and residual variances are thereby assumed to follow a log-normal distribution on the between-person level. This modeling strategy is illustrated in Figure 1. This approach is also adopted and described in the commented Stan model codes in the accompanying tutorial.

Figure A1: Modeling random innovation covariances

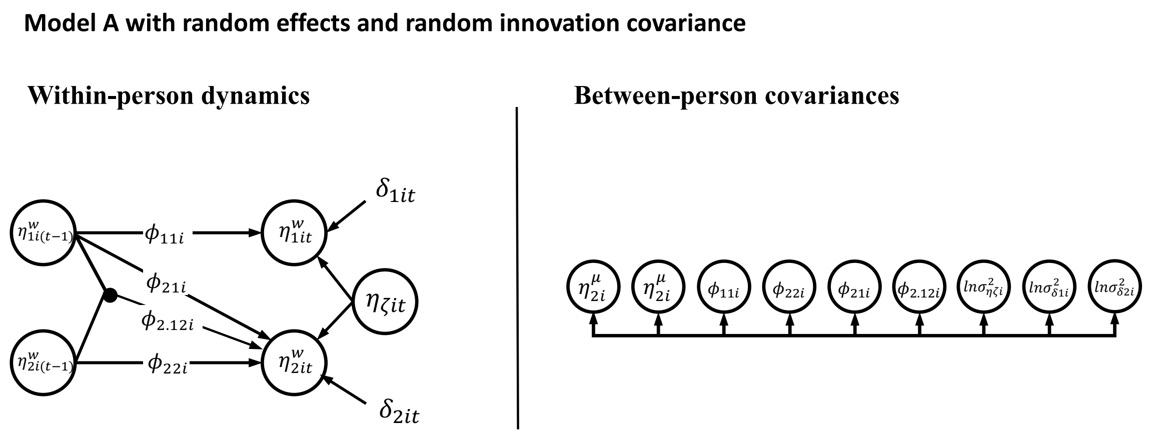

A.2 Computation of standardized within-level parameter estimates

To assess the relative strengths of the different CR relationships, researchers usually rely on standardized parameter estimates. We calculate standardized within-level moderated VAR model coefficients in line with the approach for standardization in latent interaction models outlined in Asparouhov & Muthen (2021). That is, given a latent interaction model of the form

$$\hat{\eta}_3 = \beta_1 * \eta_1 + \beta_2 * \eta_2 + \beta_3 * \eta_1 * \eta_2$$

β_1 and β_2 are standardized as usual, by dividing the respective coefficient by the standard deviation of the dependent variable and multiplying by the standard deviation of the predictor variable, e.g., for β_1 resulting in $\beta_1/SD(\eta_3) * SD(\eta_1)$. Standardizing the interaction effect is based on Wen et al. (2010), using $\sqrt{(}Var(\eta_1))\sqrt{(}Var(\eta_2))$ on the predictor side, resulting in $\beta_3/SD(\eta_3) * SD(\eta_1) * SD(\eta_2)$.

Note the following particularities when applying the standardization outlined above to the multilevel moderated VAR models in the empirical application. First, in lack of an analytical expression for the variances of the within-level latent factors η_q^w in multilevel VAR models including interaction terms (for VAR models without interactions see, e.g., Schuurman et al., 2016), the respective variances are determined empirically, based on the posterior samples of the η_q^w. Standardization is carried out within each MCMC iteration, generating a posterior distribution of the standardized effects. Second, in case of multilevel VAR models, within-person standardization is recommended for CR effects (Schuurman et al., 2016). Consequently, in random effect models, we implement the standardization based on each individuals' within-level latent factor variances and subsequently average these across persons to yield an average standardized effect. Again, this is done per MCMC iteration within the model estimation, providing a respective posterior distribution which can be used to build credibility intervals.

Appendix B

Empirical example 1: data by Blanke et al. (2020)

The following models were fitted to the dataset by Blanke et al. (2020), which were made available at https://osf.io/nvt6a/ . For descriptions of the items see Blanke et al. (2020).

Model 1: multilevel latent AR model with simultaneous (interaction) effects of rumination and mindful attention on negative affect

Figure A2: Multilevel latent AR(1) model used in the data application (model with simultaneous effects according to Blanke et al., 2020). Construct 1: mindful attention; Construct 2: negative affect; Construct 3: rumination.

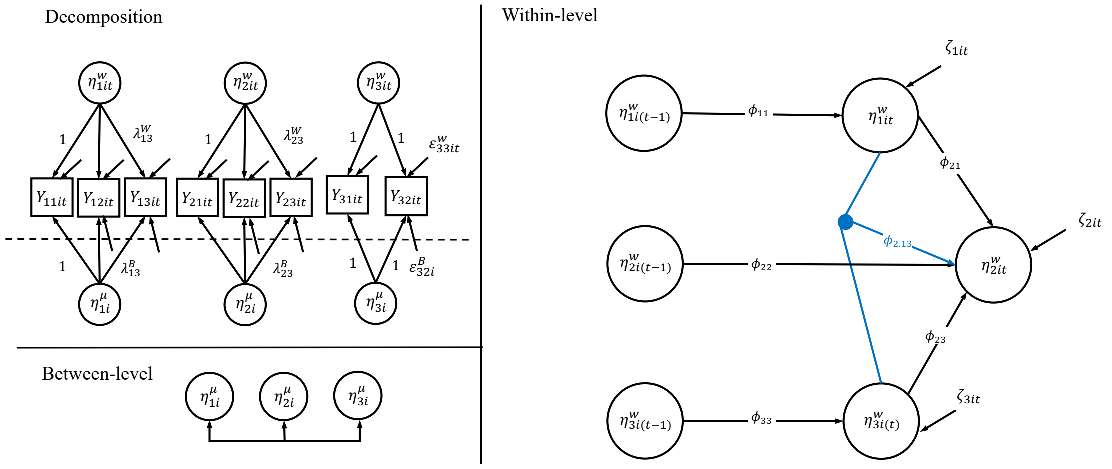

Table A1: Within-level parameter estimates of the application of the multilevel latent AR model with simultaneous (interaction) effects of mindful attention and rumination, on negative affect.

**Parameter**	**meaning**	**mean**	**2.5%**	**97.5%**
**Within-level regression parameters ϕ (unstandardized)**				
ϕ_{11}	$AR\ MF_{(t-1)} \rightarrow MF_t$	0.247	0.202	0.293
ϕ_{22}	$AR\ NA(t-1) \rightarrow NA_t$	0.230	0.187	0.273
ϕ_{33}	$AR\ RU_{(t-1)} \rightarrow RU_t$	0.346	0.299	0.393
ϕ_{21}	$CR\ MF_t \rightarrow NA_t$	-0.203	-0.246	-0.159
ϕ_{23}	$CR\ RU_t \rightarrow NA_t$	0.474	0.423	0.527
$\phi_{2.13}$	$MF_t * RU_t \rightarrow NA_t$	-0.317	-0.388	-0.254
σ_{ζ_1}	Innovation $SD\ MF$	0.949	0.908	0.991
σ_{ζ_2}	Innovation $SD\ NA$	0.548	0.503	0.592
σ_{ζ_3}	Innovation $SD\ RU$	0.821	0.784	0.858
**Standardized within-level regression parameters ϕ**				
ϕ_{21}	$CR\ MF_t \rightarrow NA_t$	-0.226	-0.273	-0.179
ϕ_{23}	$CR\ RU_t \rightarrow NA_t$	0.491	0.441	0.543
$\phi_{2.13}$	$MF_t * RU_t \rightarrow NA_t$	-0.359	-0.432	-0.292
**R^2 for the within-level latent variables**				
η_1^W	MF	.229	.182	.276
η_2^W	NA	.557	.503	.612
η_3^W	RU	.470	.421	.519
**Within-level measurement model**				
λ_{W12}	factor loading MF	0.607	0.566	0.648
λ_{W13}	factor loading MF	0.709	0.668	0.751
λ_{W22}	factor loading NA	0.865	0.824	0.906
λ_{W23}	factor loading NA	0.409	0.363	0.456
$\sigma_{\epsilon_{11}^W}$	measurement error $SD\ MF$	0.790	0.749	0.829
$\sigma_{\epsilon_{12}^W}$	measurement error $SD\ MF$	0.967	0.941	0.993
$\sigma_{\epsilon_{13}^W}$	measurement error $SD\ MF$	0.894	0.867	0.921
$\sigma_{\epsilon_{21}^W}$	measurement error $SD\ NA$	0.664	0.633	0.694
$\sigma_{\epsilon_{22}^W}$	measurement error $SD\ NA$	0.744	0.718	0.772
$\sigma_{\epsilon_{23}^W}$	measurement error $SD\ NA$	1.145	1.118	1.172
$\sigma_{\epsilon_{31}^W}$	measurement error $SD\ RU$	0.842	0.811	0.875
$\sigma_{\epsilon_{32}^W}$	measurement error $SD\ RU$	0.989	0.957	1.021
**Within-level reliabilities**				
η_{11}^W	MF	.656	.626	.686
η_{12}^W	MF	.317	.286	.349
η_{13}^W	MF	.423	.390	.456
η_{21}^W	NA	.676	.651	.701
η_{22}^W	NA	.569	.539	.600
η_{23}^W	NA	.113	.090	.137
η_{31}^W	RU	.608	.583	.633
η_{32}^W	RU	.515	.494	.536

Note. The columns mean, 2.5%, and 97.5% refer to the respective mean and quantiles of the posterior Markov-Chain Monte-Carlo samples / the posterior distribution. AR: autoregressive effect; CL: cross-regression effect; corr.: correlation; SD: standard deviation; MF: mindful attention; NA: negative affect; RU: rumination.

Table A2: Between-level parameter estimates of the application of the multilevel latent AR model with simultaneous (interaction) effects of mindful attention and rumination, on negative affect.

Parameter	meaning	mean	2.5%	97.5%
**Between-level stable trait variables**				
γ_{μ_1}	fixed effect trait MF	3.825	3.661	3.992
γ_{μ_2}	fixed effect trait NA	1.024	0.811	1.245
γ_{μ_3}	fixed effect trait RU	1.375	1.151	1.600
τ_{μ_1}	random effect SD trait MF	0.676	0.562	0.817
τ_{μ_2}	random effect SD trait NA	0.889	0.749	1.063
τ_{μ_3}	random effect SD trait RU	0.921	0.773	1.104
ρ_{μ_1,μ_2}	trait correlation $MF - RU$	-0.129	-0.365	0.118
ρ_{μ_1,μ_3}	trait correlation $MF - NA$	-0.431	-0.615	-0.218
ρ_{μ_2,μ_3}	trait correlation $RU - NA$	0.492	0.291	0.663
$\sigma_{\epsilon^B_{12}}$	residual trait SD MF	0.345	0.279	0.424
$\sigma_{\epsilon^B_{13}}$	residual trait SD MF	0.400	0.330	0.486
$\sigma_{\epsilon^B_{22}}$	residual trait SD NA	0.654	0.549	0.785
$\sigma_{\epsilon^B_{23}}$	residual trait SD NA	0.824	0.690	0.986
$\sigma_{\epsilon^B_{32}}$	residual trait SD RU	0.560	0.467	0.674
**Between-level measurement model**				
α_{12}	item intercept MF	0.689	0.122	1.245
α_{13}	item intercept MF	0.829	0.210	1.447
α_{22}	item intercept NA	0.267	0.029	0.514
α_{23}	item intercept NA	0.903	0.592	1.208
α_{32}	item intercept RU	0.403	0.264	0.544
λ_{B12}	factor loading MF	0.874	0.729	1.019
λ_{B13}	factor loading MF	0.803	0.644	0.963
λ_{B22}	factor loading NA	0.953	0.770	1.133
λ_{B23}	factor loading NA	0.694	0.464	0.925

Note. The columns mean, 2.5%, and 97.5% refer to the respective mean and quantiles of the posterior Markov-Chain Monte-Carlos samples / the posterior distribution. AR: autoregressive effect; CL: cross-regression effect; SD: standard deviation; MF: mindful attention; NA: negative affect; RU: rumination.

Model 2: Lagged multilevel latent VAR model for rumination, mindful attention, and negative affect

Figure A3: Multilevel latent moderated VAR(1) model used in the data application. Construct 1: mindful attention; Construct 2: negative affect; Construct 3: rumination.

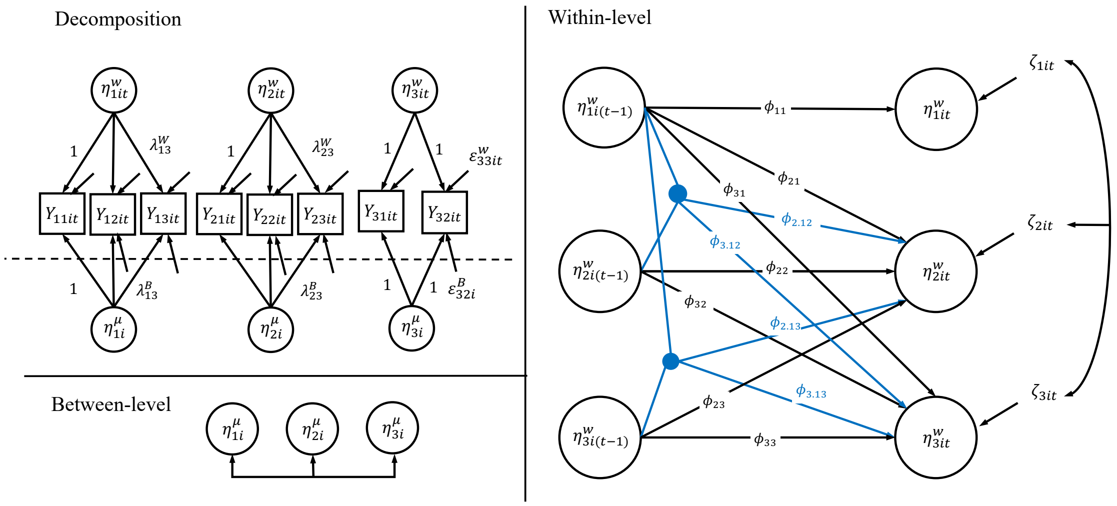

Table A3: Within-level parameter estimates of the application of the multilevel latent moderated VAR model for mindful attention, rumination, and negative affect.

Parameter	meaning	mean	2.5%	97.5%
**Within-level regression parameters ϕ (unstandardized)**				
ϕ_{11}	$AR\ MF_{(t-1)} \rightarrow MF_t$	0.238	0.192	0.284
ϕ_{22}	$AR\ NA(t-1) \rightarrow NA_t$	0.350	0.290	0.409
ϕ_{33}	$AR\ RU_{(t-1)} \rightarrow RU_t$	0.231	0.170	0.294
ϕ_{21}	$CR\ MF_{(t-1)} \rightarrow NA_t$	-0.020	-0.059	0.020
ϕ_{23}	$CR\ RU_{(t-1)} \rightarrow NA_t$	0.071	0.018	0.124
ϕ_{31}	$CR\ MF_{(t-1)} \rightarrow RU_t$	-0.045	-0.087	-0.002
ϕ_{32}	$CR\ NA_{(t-1)} \rightarrow RU_t$	0.133	0.071	0.195
$\phi_{2.12}$	$MF_{(t-1)} * NA_{(t-1)} \rightarrow NA_t$	-0.043	-0.110	0.023
$\phi_{2.13}$	$MF_{(t-1)} * RU_{(t-1)} \rightarrow NA_t$	-0.055	-0.106	-0.004
$\phi_{3.12}$	$MF_{(t-1)} * NA_{(t-1)} \rightarrow RU_t$	-0.039	-0.100	0.020
$\phi_{3.13}$	$MF_{(t-1)} * RU_{(t-1)} \rightarrow RU_t$	-0.018	-0.077	0.041
σ_{ζ_1}	Innovation $SD\ MF$	0.962	0.922	1.004
σ_{ζ_2}	Innovation $SD\ NA$	0.781	0.747	0.814
σ_{ζ_3}	Innovation $SD\ RU$	0.815	0.779	0.851
$\rho_{\zeta_{12}}$	Innov. corr. $MF - NA$	-0.324	-0.373	-0.274
$\rho_{\zeta_{13}}$	Innov. corr. $MF - RU$	-0.294	-0.345	-0.241
$\rho_{\zeta_{23}}$	Innov. corr. $NA - RU$	0.575	0.528	0.622
**Standardized within-level regression parameters ϕ**				
ϕ_{21}	$CR\ MF_{(t-1)} \rightarrow NA_t$	-0.022	-0.066	0.022
ϕ_{23}	$CR\ RU_{(t-1)} \rightarrow NA_t$	0.074	0.019	0.129
ϕ_{31}	$CR\ MF_{(t-1)} \rightarrow RU_t$	-0.048	-0.094	-0.002
ϕ_{32}	$CR\ NA_{(t-1)} \rightarrow RU_t$	0.127	0.068	0.187
$\phi_{2.12}$	$MF_{(t-1)} * NA_{(t-1)} \rightarrow NA_t$	-0.047	-0.120	0.025
$\phi_{2.13}$	$MF_{(t-1)} * RU_{(t-1)} \rightarrow NA_t$	-0.063	-0.121	-0.005
$\phi_{3.12}$	$MF_{(t-1)} * NA_{(t-1)} \rightarrow RU_t$	-0.041	-0.105	0.021
$\phi_{3.13}$	$MF_{(t-1)} * RU_{(t-1)} \rightarrow RU_t$	-0.020	-0.085	0.045
**Within-level measurement model**				
λ_{W12}	factor loading MF	0.600	0.559	0.642
λ_{W13}	factor loading MF	0.700	0.658	0.743
λ_{W22}	factor loading NA	0.872	0.830	0.914
λ_{W23}	factor loading NA	0.413	0.367	0.460
$\sigma_{\epsilon^W_{11}}$	measurement error $SD\ MF$	0.763	0.720	0.804
$\sigma_{\epsilon^W_{12}}$	measurement error $SD\ MF$	0.967	0.941	0.993
$\sigma_{\epsilon^W_{13}}$	measurement error $SD\ MF$	0.894	0.867	0.923
$\sigma_{\epsilon^W_{21}}$	measurement error $SD\ NA$	0.673	0.642	0.703
$\sigma_{\epsilon^W_{22}}$	measurement error $SD\ NA$	0.741	0.714	0.769
$\sigma_{\epsilon^W_{23}}$	measurement error $SD\ NA$	1.144	1.117	1.171
$\sigma_{\epsilon^W_{31}}$	measurement error $SD\ RU$	0.821	0.790	0.854
$\sigma_{\epsilon^W_{32}}$	measurement error $SD\ RU$	0.994	0.963	1.025

Note. The columns mean, 2.5%, and 97.5% refer to the respective mean and quantiles of the posterior Markov-Chain Monte-Carlo samples / the posterior distribution. AR: autoregressive effect; CL: cross-regression effect; corr.: correlation; SD: standard deviation; MF: mindful attention; NA: negative affect; RU: rumination.

Table A4: Between-level parameter estimates of the application of the multilevel latent moderated VAR model for mindful attention, rumination, and negative affect.

Parameter	meaning	mean	2.5%	97.5%
**Between-level stable trait variables**				
γ_{μ_1}	fixed effect trait MF	3.825	3.651	3.998
γ_{μ_2}	fixed effect trait NA	1.047	0.820	1.267
γ_{μ_3}	fixed effect trait RU	1.347	1.124	1.568
τ_{μ_1}	random effect SD trait MF	0.697	0.580	0.838
τ_{μ_2}	random effect SD trait NA	0.906	0.760	1.083
τ_{μ_3}	random effect SD trait RU	0.926	0.780	1.103
ρ_{μ_1,μ_2}	trait correlation $MF - RU$	-0.128	-0.363	0.113
ρ_{μ_1,μ_3}	trait correlation $MF - NA$	-0.459	-0.639	-0.242
ρ_{μ_2,μ_3}	trait correlation $RU - NA$	0.516	0.319	0.680
$\sigma_{\epsilon^B_{12}}$	residual trait SD MF	0.343	0.277	0.423
$\sigma_{\epsilon^B_{13}}$	residual trait SD MF	0.400	0.330	0.486
$\sigma_{\epsilon^B_{22}}$	residual trait SD NA	0.655	0.555	0.781
$\sigma_{\epsilon^B_{23}}$	residual trait SD NA	0.818	0.685	0.978
$\sigma_{\epsilon^B_{32}}$	residual trait SD RU	0.561	0.468	0.675
**Between-level measurement model**				
α_{12}	item intercept MF	0.686	0.149	1.231
α_{13}	item intercept MF	0.811	0.204	1.419
α_{22}	item intercept NA	0.272	0.029	0.512
α_{23}	item intercept NA	0.881	0.583	1.185
α_{32}	item intercept RU	0.404	0.265	0.543
λ_{B12}	factor loading MF	0.875	0.734	1.013
λ_{B13}	factor loading MF	0.808	0.653	0.961
λ_{B22}	factor loading NA	0.945	0.766	1.122
λ_{B23}	factor loading NA	0.707	0.483	0.929

Note. The columns mean, 2.5%, and 97.5% refer to the respective mean and quantiles of the posterior Markov-Chain Monte-Carlos samples / the posterior distribution. AR: autoregressive effect; CL: cross-regression effect; SD: standard deviation; MF: mindful attention; NA: negative affect; RU: rumination.

Appendix C

Empirical example 2: data by Houben et al.

Multilevel latent moderated VAR model of positive and negative affect

Table A5: Parameter estimates of the application of the multilevel latent moderated VAR model with random effects for rumination and negative affect.

		Fixed effects			Random effect SDs		
**Parameter**	**meaning**	**mean**	**2.5%**	**97.5%**	**mean**	**2.5%**	**97.5%**
μ_1	Trait RU	2.293	2.115	2.468	1.266	1.147	1.401
μ_2	Trait NA	1.252	1.152	1.353	0.713	0.644	0.790
$ln(\sigma_{\zeta_1})$	log innovation SD RU	0.678	0.584	0.772	0.676	0.613	0.747
$ln(\sigma_{\zeta_2})$	log innovation SD NA	-0.544	-0.652	-0.434	0.797	0.724	0.880
ϕ_{11}	$AR\ RU_{(t-1)} \rightarrow RU_t$	0.290	0.262	0.316	0.172	0.152	0.194
ϕ_{12}	$CR\ NA_{(t-1)} \rightarrow RU_t$	0.217	0.187	0.247	0.149	0.122	0.179
ϕ_{22}	$AR\ NA_{(t-1)} \rightarrow NA_t$	0.281	0.258	0.303	0.142	0.125	0.161
ϕ_{21}	$CR\ RU_t \rightarrow NA_t$	0.178	0.161	0.196	0.115	0.102	0.130
$\phi_{2.12}$	$RU_t * NA_{(t-1)} \rightarrow NA_t$	0.028	0.018	0.039	0.058	0.049	0.068

Note. The columns mean, 2.5%, and 97.5% refer to the respective mean and quantiles of the posterior Markov-Chain Monte-Carlos samples / the posterior distribution. AR: autoregressive effect; CL: cross-regression effect; SD: standard deviation; NA: negative affect; RU: rumination.

Figure A4: Multilevel latent moderated VAR(1) model used in the data application. Construct 1: rumination; Construct 2: negative affect.

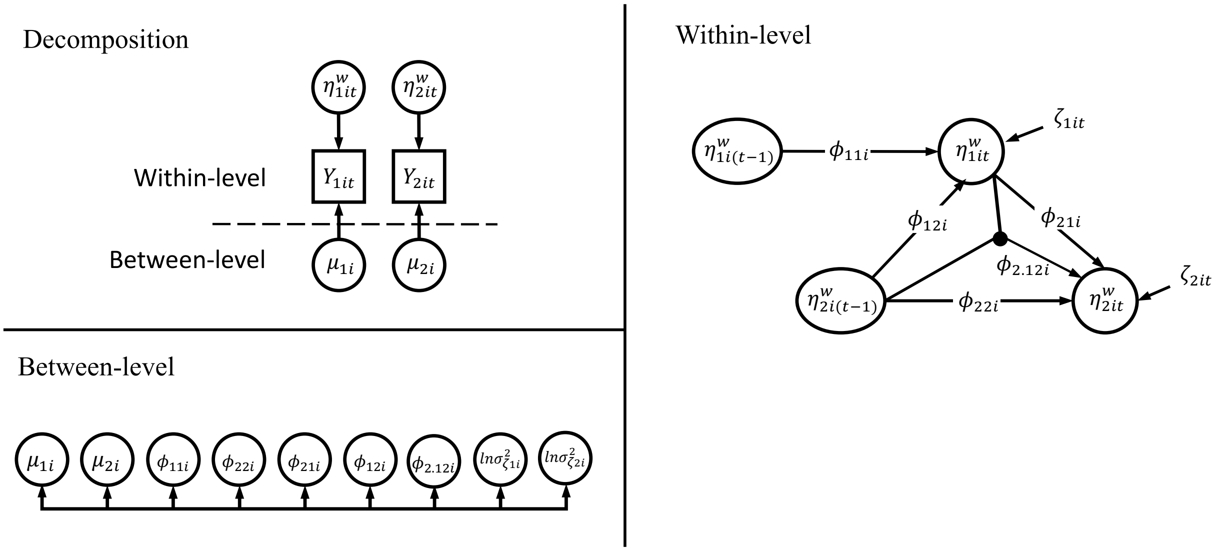

Table A6: Between-level random effect correlations in the application of the multilevel latent moderated VAR model with random effects for rumination and negative affect.

	μ_{RU}	μ_{NA}	$ln(\sigma_{\zeta_{RU}})$	$ln(\sigma_{\zeta_{NA}})$	ϕ_{11}	ϕ_{12}	ϕ_{21}	ϕ_{22}	$\phi_{2.12}$
μ_{RU}	-	[.68; .81]	[.53; .70]	[.45; .64]	[.01; .31]	[-.19; .17]	[-.22; .07]	[.02; .33]	[-.20; .14]
μ_{NA}	.750	-	[.19; .44]	[.51; .69]	[-.10; .21]	[-.28; .08]	[.09; .37]	[.07; .38]	[-.28; .05]
$ln(\sigma_{\zeta_{RU}})$	.618	.316	-	[.54; .71]	[-.12; .18]	[-.10; .26]	[-.28; .01]	[-.06; .25]	[-.18; .20]
$ln(\sigma_{\zeta_{NA}})$	.549	.603	.628	-	[-.13; .17]	[-.45; -.08]	[.15; .42]	[-.07; .25]	[-.27; .08]
ϕ_{11}	.169	.053	.029	.021	-	[-.23; .16]	[-.32; .01]	[.10; .43]	[-.06; .33]
ϕ_{12}	-.011	-.104	.085	-.269	-.038	-	[-.19; .21]	[.03; .42]	[-.16; .31]
ϕ_{21}	-.079	.236	-.137	.287	-.162	.007	-	[-.45; -.13]	[-.18; .20]
ϕ_{22}	.182	.229	.096	.090	.273	.225	-.295	-	[-.22; .15]
$\phi_{2.12}$	-.025	-.121	.012	-.094	.140	.079	.011	-.035	-

Note. The columns mean, 2.5%, and 97.5% refer to the respective mean and quantiles of the posterior Markov-Chain Monte-Carlos samples / the posterior distribution. *AR*: autoregressive effect; *CL*: cross-regression effect; *SD*: standard deviation; *NA*: negative affect; *RU*: rumination.

Appendix D

Tutorial

Motivation

When working with Stan via the `rstan`-package a typical workflow involves four steps: (1) data preparation to set up a list of data that will be passed to Stan via `R`, (2) writing the Stan model code, (3) model estimation, and (4) investigating posterior statistics and visualization of results in `R`.

Step (1) involves setting up the data in accordance to the specific Stan model. An illustration of how to prepare the data for the Stan models presented here, using simulated data sets, is provided in the accompanying R-scripts that can be accessed at: https://osf.io/bvsqy/files/osfstorage. These scripts comprise R code for preparing and saving the data and information that has to be passed to Stan for estimation, including the indexing of missing values. Furthermore, the R-scripts comprise code for the basic steps of model estimation and convergence checking via the R-packages `rstan`.

For a more detailed description of the steps that should be part of (4), we refer to existing (and comprehensive, as well as extensive) tutorials and vignettes [e.g., @gabryVisualizationBayesianWorkflow2019].

Here, we provide a brief illustration on how to implement and adapt Stan model codes to fit the multilevel latent moderated VAR models presented in the paper for their use in applied research settings. To foster the use of the models examined in the simulation studies (or variants thereof), the Stan model codes were adapted to:

1. handle unequal numbers of observations per subject,
2. handle missing values (under MCAR/MAR) in the manifest indicator variables via imputation of missing values,
3. address potential overnight-lags in the time-series data,
4. include an additional measurement model on the between-level. Note that the same factor structure on the between- as on the within-level is assumed here. However, the model codes can be easily adapted to circumvent this assumption.

A full introduction to Stan is beyond the scope of this illustration. For readers who are not familiar with Stan, we refer to the Stan documentation [Stan Development Team, -@standevelopmentteamRStanInterfaceStan2023]. A detailed tutorial on how to set up VAR(1) models can be found in @liFittingMultilevelVector2022.

Fixed Effect Model

We begin the demonstration with a model that assumes fixed dynamic parameters across subjects, with latent trait variables which are modeled as random effects (random means / intercepts). For an extension to models that include random effects for all dynamic parameters see Section 3 further below.

The data-generating model used here involves two latent variables that are measured by three manifest indicators each (multiple-indicator latent factor model). Critically, as an extension to the common (latent) VAR(1) model, the within-dynamics of the second latent construct are moderated by latent momentary levels of the first construct. The within-level dynamic model is identical to Model A in the manuscript (see Figure 1). The complete Stan model code outlined below can be accessed at: https://osf.io/bvsqy/files/osfstorage.

Stan model codes are composed of different sections that follow a specific order, which we will also use to guide through the model.

Data Block

The Stan code starts with a data declaration block, which contains information on the data passed from R to Stan which will be needed for model estimation.

```
data {
 int N;                          // number of subjects
 int N_obs;                      // total number of observations across subjects
 int N_use;                      // total number of non-first observations across subjects
 int D;                          // number of latent constructs
 int N_ind;                      // total number of manifest indicators across constructs
 array[D] int N_ind_perD;        // number of indicators per construct
 int n_random;                   // number of parameters to model as random effects

 array[N] int TP_id;                                                                  ①
 array[N] int TP_use_id;                                                              ②
 int pos_use[N,max(TP_use_id)];                                                       ③
 int pos_lag[N,max(TP_use_id)];
 vector[N_obs] y_ind[N_ind];    // array of manifest indicator values (observed variables)

 // add-on in case of missing values
 int n_miss;                     // total number of missings across indicators
 int n_miss_ind[N_ind];          // number of missings per indicator
 int pos_miss_ind[N_ind,max(n_miss_ind)]; // array of the missings' positions
}
```

① To handle unequal numbers of observations per subject, an array of length `N` needs to be passed to Stan that contains the number of available observations for each subject.

② The model code is written to flexibly account for measurements nested within days by ignoring the last observation of a day as lagged predictor of the subsequent observation (on the following day). This reduces the number of observations that can function as lagged predictors of subsequent observations or analogously the number of non-first observations (of a day) for which a preceding observation is available. Similar to `TP_id`, `TP_use_id` entails the sum of observations for which the lagged observation contains a valid predictor, separately for each subject.

③ For all observations for which a valid lagged observation exists, the position (as running beep number from 2 to `TP_id`) is stored in `pos_use` as an array with a separate row for each subject. Similarly, `pos_lag` indexes the positions of all observations that can function as lagged predictors. To achieve a symmetric matrix, all remaining elements (from `TP_id` + 1 to max(`TP_id`)) are filled up with zeros (see the associated R-code).

Transformed Data Block

Here, we create an additional vector of zeros of length D (the number of latent constructs), which will be used as mean vector for sampling the innovations from a multivariate normal distribution with mean zero. According to the Stan manual, the transformed data block can be used to declare and define any variables that do not need to be changed when running a model.

```
transformed data{
  vector[D] zeros;
  for(i in 1:D){
    zeros[i] = 0;
  }
}
```

Parameters Block

Under `parameters` all model parameters are declared.

```
parameters{
 // Dynamic parameters:
  vector[6] bmu;                     // fixed effects                                  ①
  vector<lower=0>[D] sd_noise;       // SDs of innovations (within-person level)
  vector[n_random] bs[N];            // person-specific parameters
  vector<lower=0>[n_random] sd_R;    // random effect SDs (between-person variance)
  cholesky_factor_corr[n_random] L;  // cholesky factor of the between-level          ②
                                     // random-effect correlation matrix
  cholesky_factor_corr[D] L_inno;    // cholesky factor of the correlation            ③
                                     // matrix of the innovations
```

① **Note**. The model currently includes a hard-coded number of fixed effects (here: 6). When specifying a different structural model, it is essential to adjust this value accordingly.

② The between-level variance-covariance matrix is decomposed using the Cholesky factorization method. This decomposition allows for the inclusion of separate priors for the standard deviations (SDs) of the random effect variances, denoted as `sd_R`, and the Cholesky factor of the correlation matrix (elements in the random effect correlation matrix), denoted as `L`.

③ Similarly, when modeling a constant covariance or correlation between innovations across subjects, it is also possible to decompose the prediction error variance-covariance matrix into the SDs of the innovations `sd_noise` and the Cholesky factor of the respective correlation matrix `L_inno` using the Cholesky factorization method.

```
# (parameters block continued)

 // Measurement model parameters:                                                    ①
  vector[N_ind-D] lamW;              // loadings - within-level
  vector[N_ind-D] lamB;              // loadings - between-level
  vector[N_ind-D] item_int;          // item intercepts
  vector<lower=0>[N_ind-D] sigmaB;   // residual variances - between-level
                                     // (maximum is one residual less than # factors)
  vector<lower=0>[N_ind] sigmaW;     // residual (measurement error) variances - within-level

 // Additional helper parameters:
  vector[N_obs] y_lat[D];  // array of within-level latent factor scores (etaW)
  vector[N_ind-D] Yb[N];   // between-level part of each indicator (mu)

 // handling of missings
  vector[n_miss] y_impute; // vector to store imputed values for missings
}
```

① **Note**. Loading parameters (within- and between-level), item intercepts, and SDs of between-level measurement error variances are all of length (N_ind - D) (total number of indicators across constructs minus the number of latent constructs), as they are fixed to 1 or 0 for each first indicator for identification reasons.

Model Block

Model Block I - Imputation of Missing Values

For the imputation of missing values, a new array (`N_ind` × `N_obs`) named `y_merge` is declared as a local variable (meaning that the variable can only be accessed in the respective block, which saves memory resources). Afterwards, the manifest indicator scores including missing values are added to the newly created `y_merge` and, in a loop over all indicators (`1:N_ind`), missing values will be replaced with imputed values stored in `y_impute`. Indexing of the missing values' locations on the array of observations (`y_ind`) is done

via `pos_miss_ind`, an array of length `N_ind` that holds the positions of missing values on each indicator variable. Accounting for unequal numbers of `NA`s on each indicator variable is achieved by adding the number of missing values per indicator in the data block (`n_miss_ind`). In most applications, this code section does not afford any additional adaptations (unless there are no missing values on one or more of the indicators).

```
// create combined array of observed and imputed values
vector[N_obs] y_merge[N_ind];
y_merge = y_ind;      // add observations

int p_miss = 1;       // running counter variable to index positions on y_impute
for(i in 1:N_ind){    // add imputed values for missings on each indicator
  y_merge[i,pos_miss_ind[i,1:n_miss_ind[i]]] = segment(y_impute, p_miss, n_miss_ind[i]);
  p_miss = p_miss + n_miss_ind[i];    // update counter for next indicator i+1
}
```

Model Block II - Prior Specification

Location and scale values of (hyper-)priors are all hard-coded into the model code and should be inspected and adapted carefully when applying the model in light of the data set at hand.

```
# (model block continued)
// (Hyper-)Priors  on:
bmu[1:D] ~ normal(0,4);            // fixed effects of latent trait variables, i.e., random means
sd_R[1:D] ~ cauchy(0,1);           // random effect SDs of latent trait variables, i.e., random me

bmu[(D+1):6] ~ normal(0,1);        // fixed effects of AR(s),                    ①
                                   // CR(s), and interaction effect(s)
sd_noise ~ cauchy(0,1);            // SDs of innovations
L ~ lkj_corr_cholesky(1);          // correlation matrix at the between-level
L_inno ~ lkj_corr_cholesky(1);     // correlation matrix of the innovations (within-level)
item_int ~ normal(0,10);           // indicator intercepts
lamW ~ normal(1,0.5);              // factor loadings (within-level)
lamB ~ normal(1,0.5);              // factor loadings (between-level)
sigmaB ~ cauchy(0,1);              // SDs of measurement errors
sigmaW ~ cauchy(0,1);
```

① **Note**. The number of fixed effects (i.e., 6) is again hard-coded and needs to be adjusted in case additional effects are added to the (moderated) VAR process.

Model Block III - Decomposition of Manifest Indicators

The next section of the model block illustrates the decomposition of the observed indicator variables into the between-level latent random means (latent trait factors) and the within-level latent state residuals of each variable. On each level, a separate measurement model could be specified. For model identification, the item loadings of the first indicator per construct are fixed to 1 (i.e., not included in the model as a default setting). Additionally, item intercepts and between-level residual variances of the first indicator per construct are fixed to 0.

Unless one aims to implement a different factor model structure on the between- or within-level or use an alternative parameterization, this section of code is likely to remain untouched as it flexibly adapts to varying numbers of latent constructs (`D`), indicators per dimension (`N_ind_perD`), or observations per subject (`TP_id`) when provided with the respective inputs in the `data` block.

```
# (model block continued)
// local variable that will be updated in the for-loop over subjects
int pos = 1;  // the position of each subjects first obs on the y_ind-array     ①

for (pp in 1:N) {                     // start loop over subjects
  int obs_id = TP_id[pp];             // total number of obs per person         ②
  int use_id = TP_use_id[pp];         // total number of non-first obs per person
  int time_t[use_id] = pos_use[pp,1:use_id];  // position of non-first obs per person
  int time_t_lag[use_id] = pos_lag[pp,1:use_id]; // position of lagged obs per person
  int ii = 1;         // running id of indicators (1:N_ind) start with 1 for each pp
  int kk = 1;         // running id of non-first indicators start with 1 for each pp

  // decomposition of indicator variables into between- and within-level factors
  // (including the respective measurenemt models) as well as residual variables:
  for(dd in 1:D){  // start loop over latent constructs

  // 1st indicator per construct
  segment(y_merge[ii,],pos,obs_id) ~ normal(
    bs[pp,dd] + segment(y_lat[dd,], pos, obs_id), sigmaW[ii]);
    ii = ii + 1; // update indicator id

  // remaining indicators
  for(j in 2:N_ind_perD[dd]){
    kk = ii-dd;
    Yb[pp,kk] ~ normal(item_int[kk] + lamB[kk] * bs[pp,dd], sigmaB[kk]);
    segment(y_merge[ii,],pos,obs_id) ~ normal(
      Yb[pp,kk] + lamW[kk]*segment(y_lat[dd,],pos,obs_id), sigmaW[ii]);
    ii = ii+1;
    }
  } // end loop over latent constructs
```

① A local variable starting with 1, that gets updated after each loop over subjects indicating the position of each subject's first observation on the array of observed and imputed values (`y_merge`). In combination with the `segment`-function this allows handling unequal numbers of observations per subject, slicing the vectors into the elements that belong to each subject.

② Redundant local variables with the intention to improve code readability to some extent (e.g., as allows replacing `TP_use_id[pp]` with `use_id` inside the loop over subjects).

Model Block IV - Within-Level Process

After separating the latent-mean centered within-level state residual from stable trait levels (random means), indicator intercepts, and measurement error, the dynamic process is defined using the latent within-level state factors stored in `y_lat` (η^W in the paper). Again, to improve code readability, we repeatedly declare `y_lat_id` as a local variable that holds a copy of all latent factor scores of subject `pp` to avoid reusing the `segment`-function each time it is referred to `y_lat`. The `multi_normal` sampling statement is placed on the innovations (`innos`) of each construct (`D`), which are given by the difference between the latent factor score at time t (using `time_t` that holds positions of all non-first observations of each subject) and it's predicted values based on the values at the preceding time point $t-1$ (stored in `time_t_lag`).

The loop over subjects ends after the running position indicator `pos` is updated. As a final part of the model block, the sampling statement on the person-specific trait scores is placed with the fixed effects vector `bmu`, and the Cholesky factorized elements of variance-covariance matrix (`L` and `sd_R`) entered.

```
1       # (model block continued)
2       vector[obs_id] y_lat_id[D];
3       for(dd in 1:D){
4       y_lat_id[dd,] = segment(y_lat[dd,], pos, obs_id);
5       }
6
7       vector[D] innos[use_id];             // array of innovations
8       innos[,1] = to_array_1d(y_lat_id[1,time_t] -                                    ①
9         (  bmu[3] * y_lat_id[1,time_t_lag]));
10
11      innos[,2] = to_array_1d(y_lat_id[2,time_t] -                                    ②
12        (  bmu[4] *  y_lat_id[2,time_t_lag] + bmu[5] * y_lat_id[1,time_t_lag]
13         + bmu[6] * (y_lat_id[1,time_t_lag] .* y_lat_id[2,time_t_lag])));
14
15      // sampling statements
16      innos ~ multi_normal_cholesky(zeros, diag_pre_multiply(sd_noise, L_inno));
17
18      // update position indices for next subject pp+1
19      pos = pos + obs_id;
20      } \\ end loop over subjects
21
22    // sampling statement for person-specific parameters from multivariate normal distribution
23    bs ~ multi_normal_cholesky(bmu[1:n_random], diag_pre_multiply(sd_R, L));
24
25  } // end model block
```

① Within-level latent process for construct 1. In this model, construct 1 follows a simple AR(1) process on the latent within-level.

② Within-level latent process for construct 2. In this example, a within-level interaction of latent factor scores is specified only for the second variable. See Model A in the manuscript or OSM2 for details on the model. This within-level process can be easily adapted to alternative models, by adjusting lines 8-14 above. Example codes for different within-level models are provided (for each model used in the simulation study) in OSM2.

Generated Quantities Block

As a final step, the between-level variance-covariance matrix (`bcov`), the random effects correlations (`bcorr`), and the correlation(s) between innovations (`bcorr_inn`) are generated. The values in these matrices are not sampled from a respective distribution but are calulated based on the values sampled for `L`, `L_inno`, and `sd_R` above. Furthermore, standardized values of CR and interaction effects are calculated based on SDs of the latent factor scores `y_lat` [see, @asparouhovBayesianEstimationSingle2021].

```
generated quantities {
  matrix[n_random,n_random] bcorr;     // random coefficients correlation matrix
  matrix[n_random,n_random] bcov;      // random coefficients covariance matrix
  matrix[D,D] bcorr_inn;               // correlation(s) of innovations
  vector[D] sd_etaW;                   // SD of within-level latent factors
  vector[2] bmus_std;                  // standardized fixed effects

  bcorr = multiply_lower_tri_self_transpose(L);
  bcov = quad_form_diag(bcorr, sd_R);
  bcorr_inn = multiply_lower_tri_self_transpose(L_inno);
  for(dd in 1:D){
```

```
    sd_etaW[dd] = sd(y_lat[dd,]);
  }

  // Standardization of fixed effects
  bmus_std[1] = bmu[5] / sd_etaW[2] * sd_etaW[1];  // cross-regression effects
  bmus_std[2] = bmu[6] * sd_etaW[1];               // interaction effects
}
```

Additional Random Effects

We now illustrate the necessary changes to the model code to model all parameters of the moderated VAR process as person-specific random effects (see Model A-RF in the article). The full model code can be accessed here: https://osf.io/bvsqy/files/osfstorage. Here, we only highlight the changes made to each model block.

(Transformed) Data Block

The data section of the model code remains unaltered, however, during data preparation the number of random effects `n_random` should be adjusted for the additional random effects (i.e., n_random = 9 [two latent traits, two AR-, one CR-, one interaction effect, two log-normal residual innovation variances, and a log innovation covariance]). The transformed data block can be removed from the model code as we no longer use the `multi_normal` sampling statement to estimate the prediction error matrix (i.e., innovation variances and their correlation). To model the person-specific innovation co-variances, we use a latent-variable approach as proposed by @hamakerFrontiersModelingIntensive2018; see OSM2 for a description. For an alternative approach, see @liFittingMultilevelVector2022.

Parameters Block

```
  vector[n_random] bmu;        // fixed effects                                  ①
  vector[n_random] bs[N];      // person-specific parameters
  vector[N_use] eta_cov;       // latent innovation covariance factor scores    ②

  \\ cholesky_factor_corr[D] L_inno;                                            ③
  \\ vector<lower=0>[D] sd_noise;

```

① In fully random models, the number of fixed effects equals the number of random effects `n_random`, which is added here.

② A new vector is declared to store the latent covariance factor scores of all subjects. See OSM2 for a description of this factor.

③ The lines referring to the Cholesky factorization of the correlation matrix and SDs of the innovations are removed, as these are now modeled within `eta_cov` and `bs`.

Transforemd Parameters Block

Person-specific residual variances and innovation co-variances are modeled to follow a log-normal distribution on the between-person level and hence, need to be retransformed to SDs on the normal scale before they can be entered in the model block as scale parameters in the `normal`-sampling statement.

```
transformed parameters{
  vector<lower=0>[N] sd_noise[D];  // retransformed log innovation variances to SDs
  vector<lower=0>[N] sd_inncov;    // retransformed log innovation covariances
                                    // to SD of latent innovation covariance factor
```

```
  sd_noise[1,1:N]  = sqrt(exp(to_vector(bs[1:N,3])));
  sd_noise[2,1:N]  = sqrt(exp(to_vector(bs[1:N,4])));
  sd_inncov[1:N]   = sqrt(exp(to_vector(bs[1:N,5])));
}
```

Model Block

In the priors section in the model block, we remove the priors placed on innovation variances (`sd_noise`) and the LKJ-prior (`L_inn`) on the innovation correlation, as these are no longer parameters within the model. We instead extend the hyperpriors on the fixed effects and random effects variances to include all of the dynamic parameters.

```
  // (Hyper-)Priors on:
  bmu[(D+1):n_random] ~ normal(0,1);  // fixed effects of ARs, CLs, and
                                      // log innovation (co-)variances
  (...)
  int pos_non1st = 1;   // additional running position indicator          ①

  for(pp in 1:N){       // start loop over subjects
  (...)
  // latent factors scores of innovation covariance
  vector[use_id] eta_cov_id = segment(eta_cov, pos_non1st, use_id);      ②
  // array of innovations
  vector[use_id] innos[D];                                                ③

  // decomposition of indicator variables:
  (...)

```

① An additional running index variable, `pos_non1st`, is declared to get the position of each subjects first observation on a vector of length `N_use` (in contrast, `pos` holds the index of first observations per subject on a vector of length `N_obs`).

② Similar to `y_lat`, we store latent factor scores of the innovation co-variance factor `eta_cov` for each subject in the local variable `eta_cov_id` using the `pos_non1st`-index. Note that we can not use `pos` here, because we are lacking the lagged predictor values, and thereby the residual term for (the) first observation(s) (of a day).

③ Note, that the dimension of the array are switched for more efficient vectorization.

Within-Level Process

As all parameters are allowed to vary over subjects, we no longer refer to the fixed effects stored in `bmu`, but the individual effects in `bs`. Additionally, applying the latent-variable approach for estimation of individual innovation co-variances, an additional latent factor `eta_cod_id` is included in the model formulation of both variables that captures the shared variance of innovations at each time point. Note that the direction of the factor loadings have to be specified a priori by setting either both to 1, or setting them to 1 and -1 in one of the equations, when assuming a positive or a negative innovation correlation, respectively. After accounting for the shared variance in the innovations, the remaining residual terms are assumed to be uncorrelated and hence, are given separate (independent) `normal` sampling statements with zero mean and a person-specific residual variance as indicated by `pp`. Similarly, a `normal` sampling statement is placed on the SD of the innovation covariance factor.

```
// within-level process:
(...)

innos[1,] = y_lat_id[1,time_t] - (
  bs[pp,6] * y_lat_id[1,time_t_lag] + eta_cov_id);
innos[2,] = y_lat_id[2,time_t] - (
  bs[pp,7] * y_lat_id[2,time_t_lag] + bs[pp,8] * y_lat_id[1,time_t_lag] +
  bs[pp,9] * (y_lat_id[1,time_t_lag] .* y_lat_id[2,time_t_lag])
  + eta_cov_id);

// sampling statements:
eta_cov_id ~ normal(0, sd_inncov[pp]);
innos[1,] ~ normal(0,sd_noise[1,pp]);
innos[2,] ~ normal(0,sd_noise[2,pp]);


pos = pos + obs_id;           // update position indices for next subject pp+1
pos_non1st = pos_non1st + use_id;
} // end loop over subjects
```

Generated Quantities

Standardized values of fixed effects in the random effects model are calculated as averaged within-person standardized effects, that is, by averaging over within-person standardized values of cross-random and interaction effects.

```
(...)
vector[N] sd_etaW[D];   // person-specific SDs of within-level latent factors
vector[N] bs_std[2];    // standardized individual effects
vector[2] bmu_std;      // average standardized effects
int pos;

(...)

pos = 1;
for(pp in 1:N){
  for(dd in 1:D){
  sd_etaW[dd,pp] = sd(segment(y_lat[dd,],pos,TP_id[pp]));
  }

  // Standardization of person-specific effects
  // Cross-regression effect(s)
  bs_std[1,pp] = bs[pp,8] / sd_etaW[2,pp] * sd_etaW[1,pp];
  // interaction effect(s)
  bs_std[2,pp] = bs[pp,9] * sd_etaW[1,pp];

  pos = pos + TP_id[pp]; // update counter
  }

for(i in 1:2){
bmu_std[i] = mean(bs_std[i,]);    // average standardized effects
}
```
